# NanoSim: nanopore sequence read simulator based on statistical characterization

**DOI:** 10.1093/gigascience/gix010

**Published:** 2017-02-24

**Authors:** Chen Yang, Justin Chu, René L Warren, Inanç Birol

**Affiliations:** 1 Canada’s Michael Smith Genome Science Centre, British Columbia Cancer Agency, 570 W 7th Avenue, V5Z 4S6 Vancouver, Canada; 2 Falculty of Science, University of British Columbia, Vancouver, Canada; 3 Department of Medical Genetics, University of British Columbia, Vancouver, Canada; 4 School of Computer Science, Simon Fraser University, Burnaby, Canada.

**Keywords:** nanopore sequencing, statistical modeling, sequence read simulation, NanoSim

## Abstract

**Background:** The MinION sequencing instrument from Oxford Nanopore Technologies (ONT) produces long read lengths from single-molecule sequencing – valuable features for detailed genome characterization. To realize the potential of this platform, a number of groups are developing bioinformatics tools tuned for the unique characteristics of its data. We note that these development efforts would benefit from a simulator software, the output of which could be used to benchmark analysis tools. **Results:** Here, we introduce NanoSim, a fast and scalable read simulator that captures the technology-specific features of ONT data and allows for adjustments upon improvement of nanopore sequencing technology. The first step of NanoSim is read characterization, which provides a comprehensive alignment-based analysis and generates a set of read profiles serving as the input to the next step, the simulation stage. The simulation stage uses the model built in the previous step to produce *in silico* reads for a given reference genome. NanoSim is written in Python and R. The source files and manual are available at the Genome Sciences Centre website: http://www.bcgsc.ca/platform/bioinfo/software/nanosim. **Conclusion:** In this work, we model the base-calling errors of ONT reads to inform the simulation of sequences with similar characteristics. We showcase the performance of NanoSim on publicly available datasets generated using the R7 and R7.3 chemistries and different sequencing kits and compare the resulting synthetic reads to those of other long-sequence simulators and experimental ONT reads. We expect NanoSim to have an enabling role in the field and benefit the development of scalable next-generation sequencing technologies for the long nanopore reads, including genome assembly, mutation detection, and even metagenomic analysis software.

## Background

DNA sequencing is dominated by sequencing-by-synthesis technologies, and mature next-generation sequencing (NGS) such as those from Illumina, Inc., are among the most widely adopted. In recent years, third-generation single-molecule sequencing using nanopore-based technologies has emerged, with promises of longer reads and lower cost. Launched by Oxford Nanopore Technologies (ONT) in April 2014, the MinION sequencer stands out among existing third-generation sequencing technologies due to its ability to generate ultra-long reads, albeit with high error rates. For example, the *Saccharomyces cerevisiae* dataset from Goodwin et al. (2015) has an average read length of 5473 bp, and a maximum reaching 147 bp, kb, although with low sequence identity, 64% for 1D reads and 75% for 2D reads, 1D and 2D referring to interrogation of a DNA molecule template once or twice, respectively.

Long nanopore reads hold great potential for *de novo* assembly and transcriptome analysis as they can span more repetitive regions and multiple exon junctions, or even entire transcripts. However, the error-prone reads pose new challenges to algorithm design [1]. As is the case for other sequencing platforms [2], a read simulator designed specifically for ONT reads is desirable in order to develop and benchmark new algorithms, with the aim to harness the full potential of this new sequencing platform. Currently, however, no state-of-the art DNA sequence simulator emulates the properties of ONT reads.

Here, we introduce NanoSim, a nanopore sequence read analysis and simulation pipeline. The tool analyzes ONT reads from experimental data to model read features, such as error profiles and length distributions, and uses these features to generate *in silico* reads for an input reference. We show that the statistical models NanoSim uses remain valid as the nanopore sequencing technology evolves.

## Methods

NanoSim is implemented using R for error model fitting and Python for read length analysis and simulation (Supplementary Fig. S1). The first step of NanoSim is read characterization, which provides a comprehensive alignment-based analysis and generates a set of read profiles serving as the input to the next step, the simulation stage. The simulation tool uses the model built in the previous step to produce *in silico* reads for a given reference genome. It also outputs a list of introduced errors, consisting of the position on each read, error type, and reference bases.

The modeling stage of NanoSim takes a reference and a training read set in FASTA format as input. The reads are aligned to the reference genome using LAST with tuned parameters (‘-r 1 -q 1 -a 1 -b 1’) by default, consistent with other published work [3,4]. Alternatively, the tool also allows the input of an alignment file in Multiple Alignment Format (MAF). If not unique, the best alignment of each read is chosen based on alignment length to avoid the influence of misalignments to repeat regions (Supplementary Fig. S2).

Based on alignment results, training reads are classified into two types: aligned and unaligned reads. For aligned reads, typically only a middle region can be aligned, leaving the flanking head and tail regions soft-clipped from alignments. The length distribution of these head and tail regions exhibits a multimodal pattern. The full read length distribution can be characterized by two empirical distributions: one for the length of the aligned regions, the second for the ratio of alignment lengths to read lengths. Length distributions of unaligned reads are also generated to simulate unaligned reads. The perfet flag of NanoSim can generate perfect reads with no errors, relying on the full-length distribution of aligned reads.

Sequencing errors on the aligned region share similar patterns among different datasets, which can be described by statistical mixture models [5]:

\begin{eqnarray*}
\begin{array}{l}
{\rm {Mismatch\!\!:}}\, {\it P}_m \sim \alpha _m\, \mathrm{Poisson}(\lambda _m)+(1-\alpha _m)\,\mathrm{Geometric}(p_m)\\
{\rm {Insertion\!\!:}}\, {\it P}_i \sim \alpha _i\, \mathrm{Weibull}(\lambda _i, \kappa _i)+(1-\alpha _i)\,\mathrm{Geometric}(p_i)\\
{\rm{Deletion\!\!:}}\, {\it P}_d \sim \alpha _d\, \mathrm{Weibull}(\lambda _d, \kappa _d)+(1-\alpha _d)\,\mathrm{Geometric}(p_d)\end{array}
\end{eqnarray*}

Here α_*m*/*i*/*d*_ ∈ (0, 1) are mixture parameters, *p*_*m*/*i*/*d*_ are the event probabilities in the geometric distributions, λ_*m*_ is the expected value of the Poisson distribution, and λ_*i*/*d*_ and κ_*i*/*d*_, respectively, are the scale and shape parameters of the Weibull distributions.

The mixture model describes stretches of substitution errors as being distributed according to Poisson distribution, whereas indels follow Weibull distributions. All error modes have a second component of geometric distribution, which we postulate describes stochastic noise. The parameters for mixture models are estimated during the modeling stage (Supplementary method). The model parameters and error profiles for the tested datasets are provided with the software download package and can be directly used for simulation.

During simulation, the lengths of errors are drawn from the statistical models, and the error types are determined by a Markov chain, simulating the transitional probability between two consecutive errors (Supplementary Fig. S3). Interval lengths between errors (length of matched bases) are observed to be auto-correlated, which justifies the use of a Markov chain to model consecutive correct base calls between errors (Supplementary Fig. S4).

Reads that are unaligned are more difficult to characterize. Rather than assuming them to be random sequences, we extract sequences from the reference and use an arbitrarily high error rate compared to the aligned reads. We pick the length of each error in these reads from the same mixture models as the aligned reads and randomly place them on the simulated sequence.

Another feature of NanoSim is that it is able to simulate either circular or linear genomes. A read extracted from a circular genome can start from any position and may wrap around. If the length of a read is longer than the length of the whole genome, which is unlikely but possible for a plasmid or viral genome, it will be truncated to the genome length. For a linear genome to maintain a read length distribution similar to the training profile, NanoSim will only extract reads from chromosomes that are longer than the read length.

The *k*-mer bias of ONT reads, especially the deficiency of long homopolymers, has been well studied [6]. As a DNA molecule with a stretch of homopolymer sequence traverses through a nanopore, the change in electric current is not detectable or fails to be interpreted by the base-calling algorithm, leading to a deficient representation of homopolymers longer than the number of bases that can fit in the nanopores. The *k*-mer bias mode of NanoSim compresses all homopolymers longer than n into *n*-mers (default n = 5), simulating the process of base-calling. The under- or overrepresentation of other *k*-mers is not supported in the current version of NanoSim. Admittedly, this method is oversimplistic because sequencing or base-calling errors occur more often in homopolymer regions, including 4-mer and 3-mer homopolymer sequence. However, we expect this sequencing bias to be addressed by the vendor and the scientific community in the future, given (i) past improvements of the R7.3 chemistry compared to the previous R7 chemistry (Supplementary Fig. S5), as well as ongoing improvements to the pore chemistry and (ii) the emergence of new and improved base-calling algorithms including DeepNano, which uses a recurrent neural network [7]. In this study, we confirm that the R9 2D dataset does not have the same homopolymer underrepresentation problem as the previous (R7 and R7.3) chemistries. However, we do observe the opposite, the presence of long homopolymers that do not exist in the reference genome.

Using an *Escherichia coli* dataset, it has been reported that the GC content of 2D reads is very close to the reference and that this has a minor effect on sequencing error rates [8]. In prior work, we have also observed that substitution errors are not uniform, with a weak bias toward G and C [5]. Since the underlying mechanism causing this bias is unclear, this pattern is not reflected in the NanoSim synthetic reads.

## Results and discussion

Six datasets using diffferent generations of sequencing kit were chosen for deriving the statistical models and benchmarking, including five *E. coli* datasets and one *S. cerevisiae* dataset (Table 1). Generally, 2D reads are higher quality than 1D reads and are more frequently used in downstream analyses. As such, we tested NanoSim on reads from 1D rapid kit using R9 chemistry, and 2D reads using R7, R7.3, and R9 chemistry. All tests were performed on a single machine with 8-core Intel i7-4770 CPUs @ 3.40 GHz and 8 GB total RAM.

### Speed and memory

The runtime of NanoSim scales up linearly with the number of reads (Supplementary Fig. S6), and the memory requirement depends on the length of the reference sequence. For example, the *E. coli* University of California, Santa Cruz (UCSC) dataset contains 45 049 2D reads with an average length of 7067 bp. Excluding read alignments, the characterization stage of NanoSim took 22m:32s, and the peak memory usage was 2.68 GB. Simulating 20 000 *E. coli* reads took 4m:39s; peak memory usage was 120 MB.

### Read alignments and model fitting

NanoSim conducts an alignment-based strategy to characterize base-calling errors; hence the read-to-reference mapping process is integral to simulations. As such, it would work the best with an alignment algorithm suitable for the sequencing platform. Designed to cope with long, error-prone reads, at the time of writing, LAST was the best-studied option, shown to capture the greatest proportion of mapped reads with few false positives [1]. Recently, the widely used BWA-MEM algorithm released an update designed for ONT reads with the -x ont2d option [9]. To reflect the state of the art, we choose LAST as our default aligner, and users can optionally choose BWA-MEM or other aligners and feed alignment results into NanoSim.

We observe that the error models derived from the characterization stage in our test datasets are consistent across both chemistries and organisms (Supplementary Tables S1–S3). Assessing the goodness of fit via a Kolmogorov-Smirnov test, we observed that base call error distributions were statistically identical to their fitted models using a *P* value threshold of 0.05 (Supplementary method). We note a subtle difference in alignments compared with the results derived from the LAST and BWA-MEM algorithms. For the UCSC *E. coli* dataset, LAST aligned 45 049 reads to the reference genome, while BWA-MEM aligned 45 047 reads. The average error rates calculated by LAST and BWA-MEM are 12.61% and 12.62%, respectively. Hence, the performance of both aligners on this dataset appears equivalent. Moreover, the overall error distributions obtained through NanoSim profiling are the same, and the structures of these models remain unchanged (Supplementary Fig. S7).

### Simulation results and comparison

Currently, there are simulators that could potentially simulate Nanopore-like reads, such as PBSIM [10], ReadSim [11], and FASTQSim [12]. Among these, PBSIM is designed to simulate reads from Pacific Biosciences (PacBio) sequencers, which also produce long yet error-rich reads. FASTQSim is a platform-independent simulator that can theoretically simulate any NGS dataset. ReadSim 1.6 is the only simulator that advertises the ability to simulate ONT reads [13].

Thus to evaluate the accuracy of NanoSim, we conducted comparisons only with ReadSim. In each experiment on the six datasets in Table 1, 20 000 synthetic reads were generated by NanoSim and ReadSim. ReadSim parameters were specifically tuned for each dataset (Supplementary method). Since ReadSim is not capable of simulating genomes with multiple chromosomes, for the yeast dataset we linked the yeast chromosomes with a single “N” before simulation and discarded synthetic reads containing Ns. Simulated reads were aligned back to the reference genome and analyzed using the characterization tool of NanoSim.

**Table 1:** Datasets used for benchmarking

Organism	Reference	Download source	Sequencing kit	Flow cell	Reference	Short form in
	genome			chemistry		paper
*E. coli* K12	*E. coli* str. K-12 substr. MG1655	http://dx.doi.org/10.5524/100102	SQK-MAP-002	R7	[4]	*E. coli* R7 dataset
*E. coli* K12	*E. coli* str. K-12 substr. MG1655	ENA: ERX708228, ERX708229, ERX708230, ERX708231	SQK-MAP-003 SQK-MAP-004	R7.3	[6]	*E. coli* R7.3 dataset
*E. coli* K12	*E. coli* str. K-12 substr. MG1655	ENA: ERX947749, ERX947750	SQK-MAP-005.1	R7.3	[18]	*E. coli* UCSC dataset
*E. coli* K12	*E. coli* str. K-12 substr. MG1655	http://s3.climb.ac.uk/nanopore/	Rapid 1D	R9	[19]	*E. coli* R9 1D dataset
*E. coli* K12	*E. coli* str. K-12 substr. MG1655	http://s3.climb.ac.uk/nanopore/	SQK-MAP-006	R9	[19]	*E. coli* R9 2D dataset
*S. cerevisiae* W303	*S. cerevisiae* S288C	http://schatzlab.cshl.edu/data/nanocorr/	NA	R7	[20]	Yeast dataset

ReadSim simulates read lengths through a sample-based method or a Gaussian model–based method. The sample-based method was used here and fed with the empirical lengths of all reads regardless of alignment results. After simulation, more than 99.9% of the synthetic reads produced by ReadSim can be aligned to the reference, while raw ONT datasets and NanoSim reads agree on the alignment rates, ranging from 82.83% to 99.68% for these four datasets.

**Figure 1:**
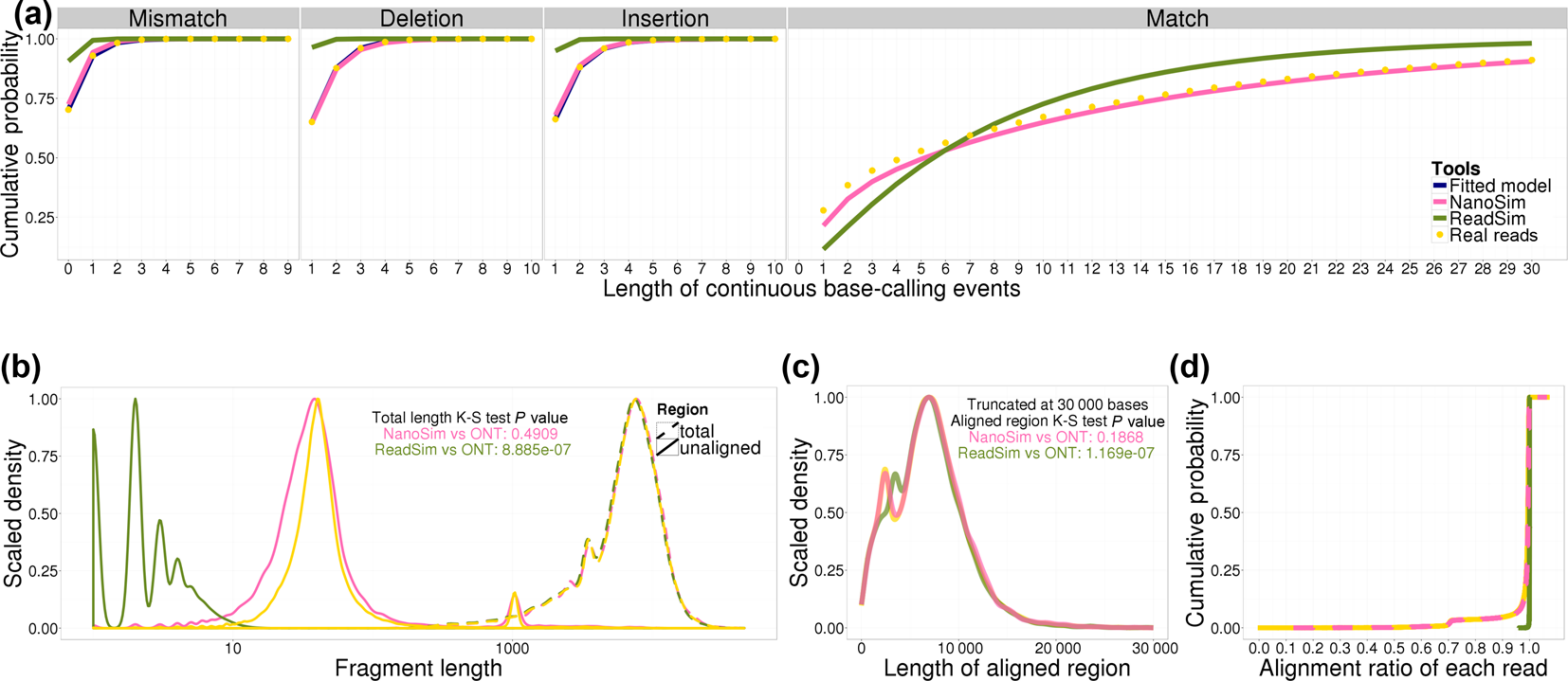
NanoSim and ReadSim simulation results compared with UCSC *E. coli* experimental reads. (**a**) The four plots on the **upper panel** are cumulative density plots of error match events and error events. (**b**) Length density plot of unaligned regions and total read lengths of aligned reads. (**c**) Length density plot of aligned regions on each read. (**d**) Cumulative density plot of the alignment ratio of each read.

The length of consecutive perfect/error bases of simulated reads were plotted together, along with their raw experimental read counterparts (Fig. 1a, Supplementary Fig. S8, Fig. S9–13a). We observed that the ReadSim reads deviate further away from experimental data because they were simulated with uniformly distributed errors and randomly chosen error length.

Statistically speaking, for all aligned reads, the lengths of the whole read and aligned regions of NanoSim reads and ONT reads are drawn from the same distributions (Fig. 1b and c, Supplementary Fig. S9–13b, S9–13c). The distribution of aligned regions also exhibits a bimodal pattern with two peaks, except for the R9 1D dataset, whereas the only length distribution ReadSim re-produces well is the full-length distribution of aligned reads on the *E. coli* R7.3 dataset (Supplementary Fig. S10b).

Since the lengths of ReadSim reads are drawn from the empirical data points directly, and more than 99.9% of ReadSim reads can be aligned, the full-length distribution of aligned ReadSim reads should prepresent the full-length distribution of all ONT reads. By comparing the full-length density of ONT and ReadSim aligned reads, we observe that the length of aligned reads and unaligned reads follows different distributions for all datasets except *E. coli* R7.3 (Supplementary Fig. S10b).

The lengths of unaligned regions are determined by the alignment ratio of each read. NanoSim performed better on *E. coli* R7 than the other three datasets, generating almost identical distributions of alignment ratio as the raw ONT reads (Supplementary Fig. S9d). This leads to similar statistical test results on the distribution of unaligned head and tail regions (Supplementary Fig. S9b). The unaligned regions on experimental ONT reads also have two peaks, and for the *E. coli* UCSC dataset, they centered at 40 bp and 1000 bp (Fig. 1b). NanoSim reads overlap with these two peaks on all six datasets, whereas ReadSim reads have much shorter unaligned regions. The head and tail regions are not profiled, and thus not recovered by ReadSim.

### *De novo* assembly of simulated reads

Testing and benchmarking new algorithms with synthetic reads is valuable tool for algorithm development as simulated reads carry the ground truth. To illustrate this, we conducted *de novo* assemblies using miniasm, an algorithm built for long reads with high error rates [14]. Dotter version 4.31 was used to compare the assemblies with the reference genome and evaluate the accuracy [15].

**Figure 2:**
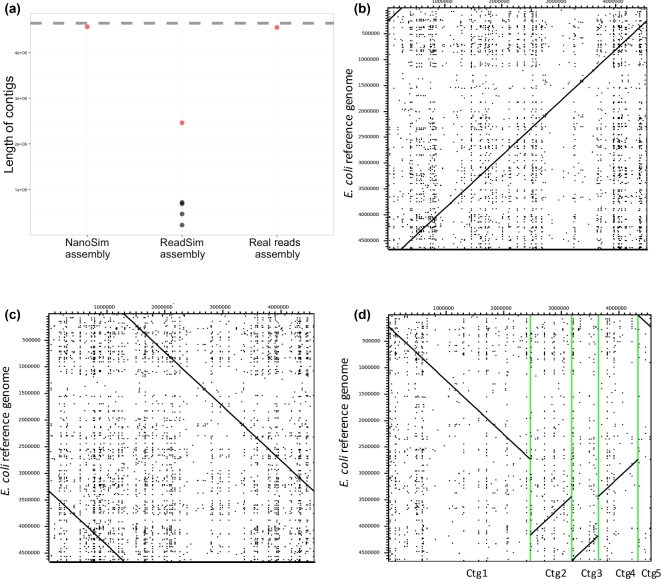
(**a**) contig sizes and N50 length of miniasm assemblies using NanoSim reads, ReadSim reads, and real reads from the UCSC dataset. The **dashed gray line** is the reference genome size and the **red dots** are contigs with N50 length. Dotter plots comparing the miniasm assembly of (**b**) experimental MinION sequence data, (**c**) NanoSim, and (**d**) ReadSim simulated reads on the x-axis to the *E. coli* K-12 MG1655 reference genome on the y-axis. The position and order of the five contigs in (d) are unclear. Accordingly, Dotter re-ordered them and aligned them along the reference genome. In this case, the x-axis represents five contigs, instead of coordinates.

Miniasm successfully assembled the UCSC dataset, and NanoSim simulated reads into one contig (Fig. 2a). Both assemblies are over 4.5 Mb in length, approaching the size of the reference genome (4.6 MB), and no large-scale misassemblies are observed (Fig. 2b and c). In contrast, ReadSim simulated reads yielded five contigs, with the largest contig reaching 2.5 Mbp. The total reconstruction matched the genome size, and the various contigs also show synteny to the reference *E. coli* K12 MG1655 genome (Fig. 2d).

## Conclusions

To our evaluation, NanoSim mimics ONT reads well, true to the major statistical features of the emerging ONT sequencing platform in terms of read length and error modes. The independent profiling module of NanoSim grants users the freedom to characterize their own ONT datasets, which are expected to evolve with nanopore sequencing technology. Yet we observe the shapes of the error models so far to hold among different datasets regardless of sequencing kit.

NanoSim will benefit the development of bioinformatics technologies for the long nanopore reads, including genome assembly, mutation detection, and metagenomic analysis software. Currently, no high-coverage human genome–size data sequenced by nanopore technologies are yet available. With the help of NanoSim, bioinformatics software developers can easily test the scalability of their tools using simulated reads. For example, NanoSim has been used for profiling and benchmarking long, error-prone reads overlapping algorithms [16]. Moreover, a mixture of *in silico* genomes simulating a microbiome will be helpful for benchmarking algorithms with application in metagenomics, including functional gene prediction, species detection, comparative metagenomics, and clinical diagnosis. As such, we expect NanoSim to have an enabling role in the field.

## Availability and requirements

Project name: NanoSim

Project home page: http://www.bcgsc.ca/platform/bioinfo/software/nanosim and https://github.com/bcgsc/nanosim

Operating system: Unix; Mac OS X

Programming lamguages: Python and R

Other requirements: LAST (tested with version 581), R (tested with version 3.2.3), Python (2.6 or above), Numpy (tested with version 1.10.1 or above)

License: GNU general public license.

## Availability of supporting data

The datasets supporting the results of this article and snapshots of the code are available in the GigaDB repository [17].

## 

### Additional files

**Additional file 1** — Supplementary method, tables, and figures

**Supplementary method:** Statistical test. **Figure S1:** Flowchart of the NanoSim profiling and simulation stages. **Figure S2:** LAST alignment performance. **Figure S3:** Transitional probabilities among different error types for *E. coli* R7 dataset. **Figure S4:** Auto-correlation of match events for *E. coli* R7 dataset. **Figure S5:***k*-mer bias of *E. coli* R7 and R7.3 datasets. **Table S1:** Mixture model parameters for mismatch. **Table S2:** Mixture model parameters for insertion. **Table S3:** Mixture model parameters for deletion. **Figure S6:** Runtime of NanoSim simulation stage on *E. coli* reference genome. **Figure S7:** Error models derived from different aligners for *E. coli* UCSC dataset. **Figure S8:** NanoSim simulation reads compared with *E. coli* UCSC experimental data and ReadSim simulated reads. **Figure S9:** NanoSim simulation results compared with *E. coli* R7 experimental reads and ReadSim simulated reads. **Figure S10:** NanoSim simulation results compared with *E. coli* R7.3 experimental reads and ReadSim simulated reads. **Figure S11:** NanoSim simulation results compared with *E. coli* R9 1D experimental reads and ReadSim simulated reads. **Figure S12:** NanoSim simulation results compared with *E. coli* R9 2D experimental reads and ReadSim simulated reads. **Figure S13:** NanoSim simulation results compared with yeast experimental reads and ReadSim simulated reads.

## Abbreviations

NGS, next-generation sequencing; ONT, Oxford Nanopore Technology; UCSC, University of California, Santa Cruz.

## Conflict of interest

The authors declare that they have no competing interests.

## Author contributions

I.B. secured the funding for the project. I.B. and C.Y. developed the NanoSim workflows. C.Y. and J.C. contributed to the development of NanoSim workflows. I.B., C.Y., J.C., and R.L.W wrote the manuscript. All authors read and approved the final manuscript.

## Acknowledgements

We thank Genome Canada, Genome British Columbia, the British Columbia Cancer Foundation, and the University of British Columbia for their financial support. This work was also partially funded by the National Institutes of Health under award number R01HG007182. The content of this work is solely the responsibility of the authors and does not necessarily represent the official views of the National Institutes of Health or other funding organizations. We thank Jared Simpson and Nick Loman for sharing unpublished work and datasets.

## Supplementary Material

GIGA-D-16-00126_Original_Submission.pdf

GIGA-D-16-00126_Revision_1.pdf

GIGA-D-16-00126_Revision_2.pdf

GIGA-D-16-00126_Revision_3.pdf

Response_to_Reviewers_Comments_Revision_1.pdf

Response_to_reviewer_comments_Original_Submission.pdf

Reviewer_1_Report_(Original_Submission).pdf

Reviewer_2_Report_(Original_Submission).pdf

Reviewer_2_Report_(Revision_1).pdf

Supplemental material
