# Supplementary material for: NanoSim: nanopore sequence read simulator based on statistical characterization
Source: Gigascience. 2017 Feb 24;6(4):1–6. doi: 10.1093/gigascience/gix010 (PMC5530317; doi:10.1093/gigascience/gix010)
Supplement: GIGA-D-16-00126_Revision_1.pdf [file gix010_GIGA-D-16-00126_Revision_1.pdf]

Manuscript Click here to download Manuscript bmc_article.tex

TECHNICAL NOTE

NanoSim: nanopore sequence read simulator based on statistical characterization

Chen Yang[1,2], Justin Chu[1,2], René L Warren[1] and Inanç Birol[1,3,4*]

[*]Correspondence: ibirol@bcgsc.ca
[1]Canada's Michael Smith Genome Science Centre, British Columbia Cancer Agency, 570 W 7th Avenue, V5Z 4S6 Vancouver, Canada
Full list of author information is available at the end of the article

Abstract

Background: The MinION sequencing instrument from Oxford Nanopore Technologies (ONT) produces long read lengths from single-molecule sequencing – valuable features for detailed genome characterization. To realize the potential of this platform, a number of groups are developing bioinformatics tools tuned for the unique characteristics of its data. We note that these development efforts would benefit from a simulator software, output of which could be used to benchmark analysis tools.

Findings: Here, we introduce NanoSim, a fast and scalable read simulator that captures the technology-specific features of ONT data, and allows for adjustments upon improvement of nanopore sequencing technology. The first step of NanoSim is read characterization, which provides a comprehensive alignment-based analysis, and generates a set of read profiles serving as the input to the next step, the simulation stage. The simulation stage uses the model built in the previous step to produce *in silico* reads for a given reference genome. NanoSim is written in Python and R. The source files and manual are available at the Genome Sciences Centre website: http://www.bcgsc.ca/platform/bioinfo/software/nanosim

Conclusion: In this work, we model the base-calling errors of ONT reads to inform the simulation of sequences with similar characteristics. We showcase the performance of NanoSim on publicly available datasets generated using the R7 and R7.3 chemistries and different sequencing kits and compare the resulting, synthetic reads, to that of other long sequence simulators and experimental ONT reads. We expect NanoSim to have an enabling role in the field and benefit the development of scalable NGS technologies for the long nanopore reads, including genome assembly, mutation detection, and even metagenomic analysis software.

Keywords: Nanopore sequencing; statistical modeling; sequence read simulation; NanoSim

Findings

Background

DNA sequencing is dominated by sequencing-by-synthesis technologies, and mature next generation systems (NGS) such as those from Illumina Inc. are amongst the most widely adopted. In recent years, third generation single molecule sequencing using nanopore-based technologies have emerged, with promises of longer reads and lower cost. Launched by Oxford Nanopore Technologies (ONT) in April 2014, the MinION sequencer stands out among existing third generation sequencing technologies due to its ability to generate ultra-long reads, albeit with high error rates. For example, the *S. cerevisiae* dataset from Goodwin *et al.* (2015) has an average

read length of 5,473 bp, and maximum reaching 147 kbp, although with low sequence identity, 64% for 1D reads and 75% for 2D reads, 1D and 2D referring to interrogation of a DNA molecule template once or twice, respectively.

Long nanopore reads hold great potential for *de novo* assembly and transcriptome analysis as they can span more repetitive regions and multiple exon junctions, or even entire transcripts. However, the error-prone reads pose new challenges to algorithm design [1]. As it is the case for other sequencing platforms [2], a read simulator designed specifically for ONT reads is desirable in order to develop and benchmark new algorithms, with the aim to harness the full potential of this new sequencing platform. Currently, however, no state-of-the art DNA sequence simulator emulates the properties of ONT reads.

Here, we introduce NanoSim, a nanopore sequence read analysis and simulation pipeline. The tool analyzes ONT reads from experimental data to model read features, such as error profiles and length distributions, and uses these features to generate *in silico* reads for an input reference. We show that the statistical models NanoSim uses remain valid as the nanopore sequencing technology evolves.

Methods

NanoSim is implemented using R for error model fitting, and Python for read length analysis and simulation (Supplementary Fig. S1). The first step of NanoSim is read characterization, which provides a comprehensive alignment-based analysis, and generates a set of read profiles serving as the input to the next step, the simulation stage. The simulation tool uses the model built in the previous step to produce *in silico* reads for a given reference genome. It also outputs a list of introduced errors, consisting of the position on each read, error type and reference bases.

The modeling stage of NanoSim takes a reference and a training read set in FASTA format as input. The reads are aligned to the reference genome using LAST with tuned parameters (`'-r 1 -q 1 -a 1 -b 1'`) by default, consistent with other published work [3, 4]. Alternatively, the tool also allows the input of an alignment file in MAF format. If not unique, the best alignment of each read is chosen based on alignment length to avoid the influence of mis-alignments to repeat regions (Supplementary Fig. S2).

Based on alignment results, training reads are classified into two types: aligned and unaligned reads. For aligned reads, typically only a middle region can be aligned, leaving the flanking head and tail regions soft-clipped from alignments. The length distribution of these head and tail regions exhibits a multimodal pattern. The full read length distribution can be characterized by two empirical distributions: one for the length of the aligned regions, the second for the ratio of alignment lengths to read lengths. Length distributions of unaligned reads are also generated to simulate unaligned reads. The `perfet` flag of NanoSim can generate perfect reads with no errors, relying on the full-length distribution of aligned reads.

Sequencing errors on the aligned region share similar patterns among different datasets, which can be described by statistical mixture models [5]:

$$\begin{aligned}
&\text{Mismatch}: && P_m \sim \alpha_m \,\text{Poisson}(\lambda_m) + (1-\alpha_m)\,\text{Geometric}(p_m)\\
&\text{Insertion}: && P_i \sim \alpha_i \,\text{Weibull}(\lambda_i, \kappa_i) + (1-\alpha_i)\,\text{Geometric}(p_i)\\
&\text{Deletion}: && P_d \sim \alpha_d \,\text{Weibull}(\lambda_d, \kappa_d) + (1-\alpha_d)\,\text{Geometric}(p_d)
\end{aligned}$$

Here $\alpha_{m/i/d} \in (0, 1)$ are mixture parameters, $p_{m/i/d}$ are the event probabilities in the geometric distributions, λ_m is the expected value of the Poisson distribution, and $\lambda_{i/d}$ and $\kappa_{i/d}$, respectively, are the scale and shape parameters of the Weibull distributions.

The mixture model describes stretches of substitution errors as being distributed according to Poisson distribution, whereas indels following Weibull distributions. All error modes have a second component of geometric distribution, which we postulate describes stochastic noise. The parameters for mixture models are estimated during the modelling stage (Supplementary Method Section). The model parameters and error profiles for the tested datasets are provided with the software download package, and can be directly used for simulation.

During simulation, the lengths of errors are drawn from the statistical models, and the error types are determined by a Markov chain, simulating the transitional probability between two consecutive errors (Supplementary Fig. S3). Interval lengths between errors (length of matched bases) are observed to be auto-correlated, and justifies the use of a Markov chain to model consecutive correct base calls between errors (Supplementary Fig. S4).

Reads that are unaligned are more difficult to characterize. Rather than assuming them to be random sequences, we extract sequences from the reference, and use an arbitrarily high error rate compared to the aligned reads. We pick the length of each error in these reads from the same mixture models as the aligned reads, and randomly place them on the simulated sequence.

Another feature of NanoSim is that it is able to simulate either circular or linear genomes. A read extracted from a circular genome can start from any position and may wrap around. If the length of a read is longer than the length of the whole genome, which is unlikely but possible for a plasmid or viral genome, it will be truncated to the genome length. For a linear genome to maintain a read length distribution similar to the training profile, NanoSim will only extract reads from chromosomes that are longer than the read length.

The k-mer bias of ONT reads, especially the deficiency of long homopolymers, has been well-studied [6]. As a DNA molecule with a stretch of homopolymer sequence traverses through a nanopore, the change in electric current is not detectable or fails to be interpreted by the base-calling algorithm, leading to a deficient representation of homopolymers longer than the number of bases that can fit in the nanopores. The k-mer bias mode of NanoSim compresses all homopolymers longer than n into n-mers (default n=5), simulating the process of base-calling. The under or over representation of other k-mers is not supported in the current version of NanoSim. Admittedly, this method is oversimplistic, because sequencing or basecalling errors occur more often in homopolymer regions, including 4-mer and 3-mer homopolymer sequence. However, we expect this sequencing bias to be addressed by the vendor in the future, given by two facts: (1) the improvement of the R7.3 chemistry compared to the previous R7 chemistry (Supplementary Fig. S5); (2) 2D reads from R9 chemistry does not have homopolymer underrepresentation problem, which is interpreted by a new basecalling algorithm using recurrent neural network.

Using an *E. coli* dataset, it has been reported that the GC content of 2D reads is very close to the reference, and that this has a minor effect on sequencing error rates

[7]. In prior work, we have also observed that substitution errors are not uniform, with a weak bias towards G and C [5]. Since the underlying mechanism causing this bias is unclear, this pattern is not reflected in the NanoSim synthetic reads.

Results and Discussion

Six datasets using diffferent generations of sequencing kit were chosen for deriving the statistical models and benchmarking, including five *E. coli* datasets and one *S. cerevisiae* dataset (Table1). Generally, 2D reads have higher quality than 1D reads, and are more frequently used in downstream analyses. As such, we tested NanoSim on reads from 1D rapid kit using R9 chemistry, and 2D reads using R7, R7.3, and R9 chemistry. All tests were performed on a single machine with 8-core Intel i7-4770 CPUs @ 3.40GHz and 8 GB total RAM.

Speed and memory

The runtime of NanoSim scales up linearly with the number of reads (Supplementary Fig. S6), and the memory requirement depends on the length of the reference sequence. For example, the *E. coli* UCSC dataset contains 45,049 2D reads with an average length of 7,067 bp. Excluding read alignments, the characterization stage of NanoSim took 22m:32s, and the peak memory usage was 2.68 GB. Simulating 20,000 *E. coli* reads took 4m:39s; peak memory usage was 120 MB.

Read alignments and model fitting

NanoSim conducts an alignment-based strategy to characterize base call errors, hence read-to-reference mapping process is integral to simulations. As such, it would work the best with an alignment algorithm suitable for the sequencing platform. Designed to cope with long, error-prone reads, at the time of writing LAST is the best studied option shown to capture the greatest proportion of mapped reads with few false positives [1]. Recently, the widely used BWA-MEM algorithm released an update designed for ONT reads with the `-x ont2d` option [8]. To reflect the state-of-the-art, we choose LAST as our default aligner, and users can optionally choose BWA-MEM or other aligners and feed alignment result into NanoSim.

We observe that the error models derived from the characterization stage in our test datasets are consistent across both chemistries and organisms (Supplementary Tables S1-S3). Assessing the goodness of fit via a Kolmogorov–Smirnov test, we observed that base call error distributions were statistically identical to their fitted models using a p-value threshold of 0.05 (Supplementary Method Section). We note subtle difference in alignments compared with the results derived from LAST and BWA-MEM algorithms. For the UCSC *E. coli* dataset, LAST aligned 45,049 reads to the reference genome, while BWA-MEM aligned 45,047 reads. The average error rates calculated by LAST and BWA-MEM are 12.61% and 12.62%, respectively. Hence, the performance of both aligners on this dataset appears equivalent. Moreover, the overall error distributions obtained through NanoSim profiling are the same, and the structures of these models remain unchanged (Supplementary Fig. S7).

Simulation results and comparison

Currently, there are simulators that could potentially simulate Nanopore-like reads, such as PBSIM [9], ReadSim [10] and FASTQSim [11]. Among these, PBSIM is designed to simulate reads from Pacific Biosciences (PacBio) sequencers, which also produce long, yet error-rich reads. FASTQSim is a platform-independent simulator that can theoretically simulate any NGS datasets. ReadSim 1.6 is the only simulator, which advertises the ability to simulate ONT reads [12].

Thus to evaluate the accuracy of NanoSim, we conducted comparisons only with ReadSim. In each experiment on the six datasets in Table 1, 20,000 synthetic reads were generated by NanoSim and ReadSim. ReadSim parameters were specifically tuned for each dataset (Supplementary Method Section). Since ReadSim is not capable of simulating genomes with multiple chromosomes, for the yeast dataset we linked the yeast chromosomes with a single “N” in between before simulation, and discarded synthetic reads containing “N”s. Simulated reads were aligned back to the reference genome and analyzed using the characterization tool of NanoSim.

ReadSim simulates read lengths through a sample-based method or a Gaussian-model-based method. The sample-based method was used here and fed with the empirical lengths of all reads regardless of alignment results. After simulation, over 99.9% synthetic reads produced by ReadSim can be aligned to the reference, while raw ONT datasets and NanoSim reads agree on the alignment rates ranging from 82.83% to 99.68% for these four datasets.

The length of consecutive perfect/error bases of simulated reads were plotted together along with their raw experimental read counterparts (Fig. 1A, Supplementary Fig. S8, Fig. S9-13A). We observed that the ReadSim reads deviate further away from experimental data because they were simulated with uniformly distributed errors and randomly chosen error length.

Statistically speaking, for all aligned reads, the lengths of the whole read and aligned regions of NanoSim reads and ONT reads are drawn from the same distributions (Fig. 1B, 1C, Supplementary Fig. S9-13B, S9-13C). The distribution of aligned regions also exhibits bimodal pattern with two peaks except for R9 1D dataset. Whereas, the only length distribution ReadSim re-produces well is the full length distribution of aligned reads on *E. coli* R7.3 dataset (Supplementary Fig. S10B).

Since the lengths of ReadSim reads are drawn from the empirical data points directly, and over 99.9% ReadSim reads can be aligned, the full-length distribution of aligned ReadSim reads should prepresent the full-length distribution of all ONT reads. By comparing the full length density of ONT and ReadSim aligned reads, we observe that the length of aligned reads and unaligned reads follow different distributions for all datasets except *E. coli* R7.3 (Supplementary Fig. S10B).

The lengths of unaligned regions are determined by the alignment ratio of each read. NanoSim performed better on *E. coli* R7 than the other three datasets, generating almost identical distributions of alignment ratio as the raw ONT reads (Supplementary Fig. S9D). This leads to similar statistical test results on the distribution of unaligned head and tail regions (Supplementary Fig. S9B). The unaligned regions on experimental ONT reads also have two peaks, and for *E. coli* UCSC dataset, they centered at 40 bp and 1000 bp (Fig. 1B). NanoSim reads overlap with these two peaks on all six datasets, whereas ReadSim reads have much

shorter unaligned regions. The head and tail regions are not profiled and thus not recovered by ReadSim.

de novo assembly of simulated reads

Testing and benchmarking new algorithms with synthetic reads is valuable tool for algorithm development, as simulated reads carry the ground truth. To illustrate this, we conducted *de novo* assemblies using miniasm, an algorithm built for long reads with high error rates [13]. Dotter version 4.31 was used to compare the assemblies with the reference genome and evaluate the accuracy [14].

Miniasm successfully assembled the UCSC dataset, and NanoSim simulated reads into one contig (Fig. 2A). Both assemblies are over 4.5 Mb in length, approaching the size of the reference genome (4.6 MB), and no large-scale misassemblies are observed (Fig. 2B, C). In contrast, ReadSim simulated reads yielded 5 contigs, with the largest contig reaching 2.5 Mbp. The total reconstruction matched the genome size and the various contigs also show synteny to the reference *E. coli* K12 MG1655 genome (Fig. 2D).

Conclusions

To our evaluation, NanoSim mimics ONT reads well, true to the major statistical features of the emerging ONT sequencing platform, in terms of read length and error modes. The independent profiling module of NanoSim grants users the freedom to characterize their own ONT datasets, which are expected to evolve with the nanopore sequencing technology. Yet, we observe the shapes of the error models so far to hold among different datasets regardless of sequencing kit.

NanoSim will benefit the development of bioinformatics technologies for the long nanopore reads, including genome assembly, mutation detection, and metagenomic analysis software. Currently, no high-coverage human genome-size data sequenced by nanopore technologies are yet available. With the help of NanoSim, bioinformatics software developers can easily test the scalability of their tools using simulated reads. For example, NanoSim has been used for profiling and benchmarking long, error-prone reads overlapping algorithms [15]. Moreover, a mixture of *in silico* genomes simulating a microbiome will be helpful for benchmarking algorithms with application in metagenomics, including functional gene prediction, species detection, comparative metagenomics, clinical diagnosis. As such, we expect NanoSim to have an enabling role in the field.

Availability and Requirements

project name: NanoSim

Project home page: http://www.bcgsc.ca/platform/bioinfo/software/nanosim and https://github.com/bcgsc/NanoSim

Operating system: Unix; Mac OS X

Programming lamguages: Python and R

Other requirements: LAST (Tested with version 581), R (Tested with version 3.2.3), Python (2.6 or above), Numpy (Tested with version 1.10.1 or above)

License: GNU General Public License - GPL.

Abbreviations

ONT: Oxford Nanopore Technology **NGS:** Next generation sequencing

Competing interests

The authors declare that they have no competing interests.

Author's contributions

IB secured the funding for the project. IB and CY developed the NanoSim workflows. CY and JC contributed to the development of Galaxy-M workflows. IB, CY, JC and RLW wrote the manuscript. All authors read and approved the final manuscript.

Acknowledgements

We thank Genome Canada, Genome British Columbia, British Columbia Cancer Foundation, and University of British Columbia for their financial support. The work is also partially funded by the National Institutes of Health under Award Number R01HG007182. The content of this work is solely the responsibility of the authors, and does not necessarily represent the official views of the National Institutes of Health or other funding organizations. We thank Jared Simpson and Nick Loman for sharing unpublished works and datasets.

Author details

[1]Canada's Michael Smith Genome Science Centre, British Columbia Cancer Agency, 570 W 7th Avenue, V5Z 4S6 Vancouver, Canada. [2]Falculty of Science, University of British Columbia, Vancouver, Canada. [3]Department of Medical Genetics, University of British Columbia, Vancouver, Canada. [4]School of Computer Science, Simon Fraser University, Burnaby, Canada.

References

1. Jain M, Fiddes IT, Miga KH, Olsen HE, Paten B, Akeson M. Improved data analysis for the MinION nanopore sequencer. Nature methods. 2015;12(4):351–356.
2. Hu X, Yuan J, Shi Y, Lu J, Liu B, Li Z, et al. pIRS: Profile-based Illumina pair-end reads simulator. Bioinformatics. 2012;28(11):1533–1535.
3. Frith MC, Hamada M, Horton P. Parameters for accurate genome alignment. BMC bioinformatics. 2010;11(1):1.
4. Quick J, Quinlan AR, Loman NJ. A reference bacterial genome dataset generated on the MinION™ portable single-molecule nanopore sequencer. Gigascience. 2014;3(1):1.
5. Warren RL, Yang C, Vandervalk BP, Behsaz B, Lagman A, Jones SJ, et al. LINKS: Scalable, alignment-free scaffolding of draft genomes with long reads. GigaScience. 2015;4(1):1.
6. Loman NJ, Quick J, Simpson JT. A complete bacterial genome assembled de novo using only nanopore sequencing data. Nature methods. 2015;12(8):733–735.
7. Karlsson E, Lärkeryd A, Sjödin A, Forsman M, Stenberg P. Scaffolding of a bacterial genome using MinION nanopore sequencing. Scientific Reports. 2015;5.
8. Li H. Aligning sequence reads, clone sequences and assembly contigs with BWA-MEM. arXiv preprint arXiv:13033997. 2013;.
9. Ono Y, Asai K, Hamada M. PBSIM: PacBio reads simulator—toward accurate genome assembly. Bioinformatics. 2013;29(1):119–121.
10. Lee H, Gurtowski J, Yoo S, Marcus S, McCombie WR, Schatz M. Error correction and assembly complexity of single molecule sequencing reads. BioRxiv. 2014;p. 006395.
11. Shcherbina A. FASTQSim: platform-independent data characterization and in silico read generation for NGS datasets. BMC research notes. 2014;7(1):1.
12. Escalona M, Rocha S, Posada D. A comparison of tools for the simulation of genomic next-generation sequencing data. Nature Reviews Genetics. 2016;17(8):459–469.
13. Li H. Minimap and miniasm: fast mapping and de novo assembly for noisy long sequences. Bioinformatics. 2016;p. btw152.
14. Sonnhammer EL, Durbin R. A dot-matrix program with dynamic threshold control suited for genomic DNA and protein sequence analysis. Gene. 1995;167(1):GC1–GC10.
15. Chu J, Mohamadi H, Warren RL, Yang C, Birol I. Innovations and challenges in detecting long read overlaps: an evaluation of the state-of-the-art. Bioinformatics. 2016;p. btw811.
16. Ip CL, Loose M, Tyson JR, de Cesare M, Brown BL, Jain M, et al. MinION Analysis and Reference Consortium: Phase 1 data release and analysis. F1000Research. 2015;4:1075.
17. Loman NJ. Nanopore R9 rapid run data release; 2016. [Online; accessed 05-Dec-2016]. http://lab.loman.net/2016/07/30/nanopore-r9-data-release/.
18. Goodwin S, Gurtowski J, Ethe-Sayers S, Deshpande P, Schatz MC, McCombie WR. Oxford Nanopore sequencing, hybrid error correction, and de novo assembly of a eukaryotic genome. Genome research. 2015;25(11):1750–1756.

Figure 1 NanoSim and ReadSim simulation results compared with UCSC E. coli experimental reads. (A) The four plots on the upper panel are cumulative density plots of error match events and error events. (B) Length density plot of unaligned regions and total read lengths of aligned reads. (C) Length density plot of aligned regions on each read. (D) Cumulative density plot of the alignment ratio of each read.

Figure 2 Tool comparison in de novo assembly. (A) Contig sizes and N50 length of miniasm assemblies using NanoSim reads, ReadSim reads and real reads from the UCSC dataset. The dashed gray line is the reference genome size and the red dots are contigs with N50 length. Dotter plots comparing the miniasm assembly of (B) experimental MinION sequence data, (C) NanoSim and (D) ReadSim simulated reads on the x-axis to the *E. coli* K-12 MG1655 reference genome on the y-axis. The position and order of the five contigs in (D) are unclear. Accordingly, Dotter re-ordered them and aligned them along the reference genome. In this case, the x-axis represents five contigs, instead of coordinates

Table 1 Datasets used for benchmarking

Organism	Reference genome	Download source	Sequencing kit	Flow cell chemistry	Reference	Short form in paper
E. coli K12	*E. coli* str. K-12 substr. MG1655	http://gigadb.org/dataset/100102	SQK-MAP-002	R7	[4]	*E. coli* R7 dataset
E. coli K12	*E. coli* str. K-12 substr. MG1655	ENA: ERX708228, ERX708229, ERX708230, ERX708231	SQK-MAP-003 SQK-MAP-004	R7.3	[6]	*E. coli* R7.3 dataset
E. coli K12	*E. coli* str. K-12 substr. MG1655	ENA: ERX947749, ERX947750	SQK-MAP-005.1	R7.3	[16]	*E. coli* UCSC dataset
E. coli K12	*E. coli* str. K-12 substr. MG1655	http://s3.climb.ac.uk/nanopore/	Rapid 1D	R9	[17]	*E. coli* R9 1D dataset
E. coli K12	*E. coli* str. K-12 substr. MG1655	http://s3.climb.ac.uk/nanopore/	SQK-MAP-006	R9	[17]	*E. coli* R9 2D dataset
S. cerevisiae W303	*S. cerevisiae* S288C	http://schatzlab.cshl.edu/data/nanocorr/	NA	R7	[18]	Yeast dataset

Figures
Tables
Additional Files
Additional file 1 — Supplementary method, tables and figures
Supplementary method: Statistical test. **Figure S1.** Flowchart of the Nanosim profiling and simulation stages. **Figure S2.** LAST alignment performance. **Figure S3.** Transitional probabilities among different error type for *E. coli* R7 dataset. **Figure S4.** Auto-correlation of match events for *E. coli* R7 dataset. **Figure S5.** *k*-mer bias of *E. coli* R7 and R7.3 datasets. **Table S1.** Mixture model parameters for mismatch. **Table S2.** Mixture model parameters for insertion. **Table S3.** Mixture model parameters for deletion. **Figure S6.** Runtime of NanoSim simulation stage on *E. coli* reference genome. **Figure S7.** Error models derived from different aligners for *E. coli* UCSC dataset. **Figure S8.** NanoSim simulation reads compared with *E. coli* UCSC experimental data and ReadSim simulated reads. **Figure S9.** NanoSim simulation results compared with *E. coli* R7 experimental reads and ReadSim simulated reads. **Figure S10.** NanoSim simulation results compared with *E. coli* R7.3 experimental reads and ReadSim simulated reads. **Figure S11.** NanoSim simulation results compared with *E. coli* R9 1D experimental reads and ReadSim simulated reads. **Figure S12.** NanoSim simulation results compared with *E. coli* R9 2D experimental reads and ReadSim simulated reads. **Figure S13.** NanoSim simulation results compared with yeast experimental reads and ReadSim simulated reads.

Figure1

Click here to download Figure Figure1.pdf

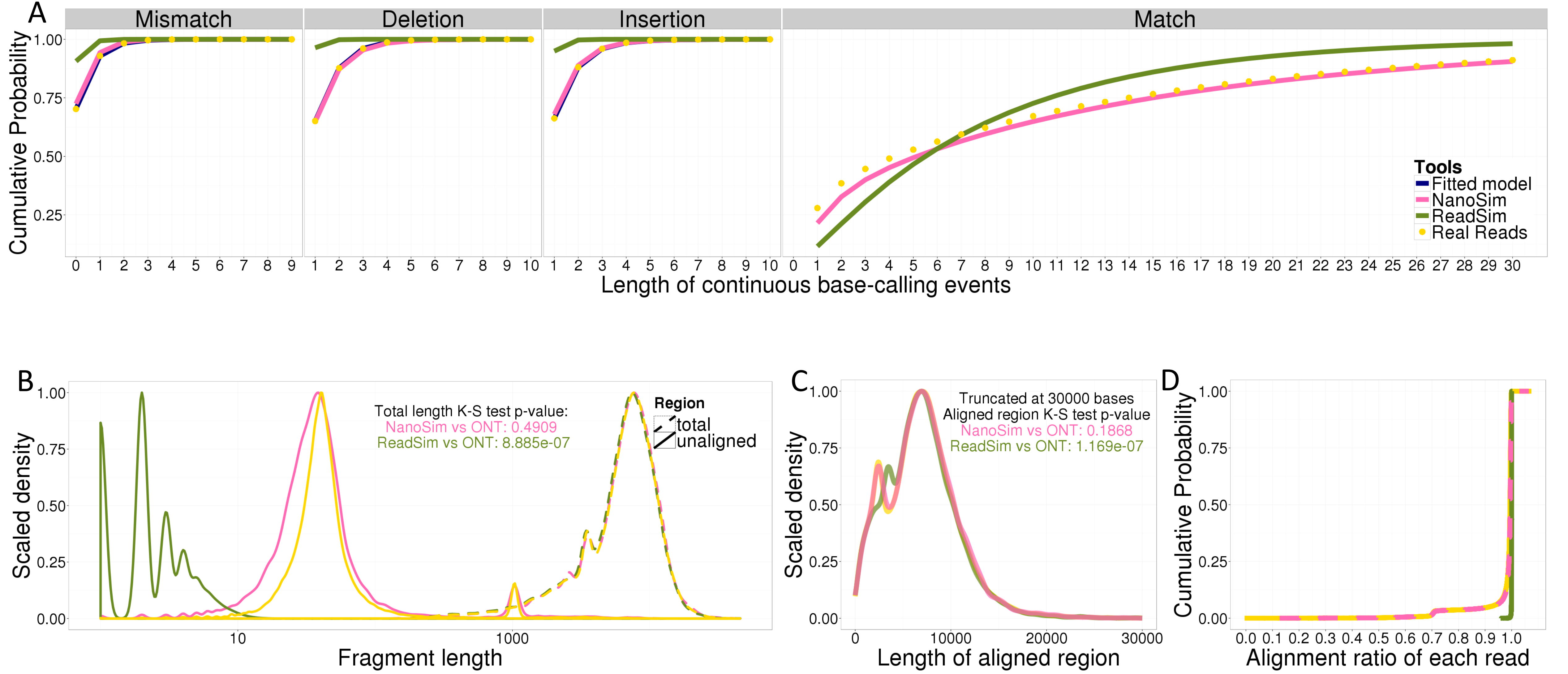

Figure2

Click here to download Figure Figure2.pdf

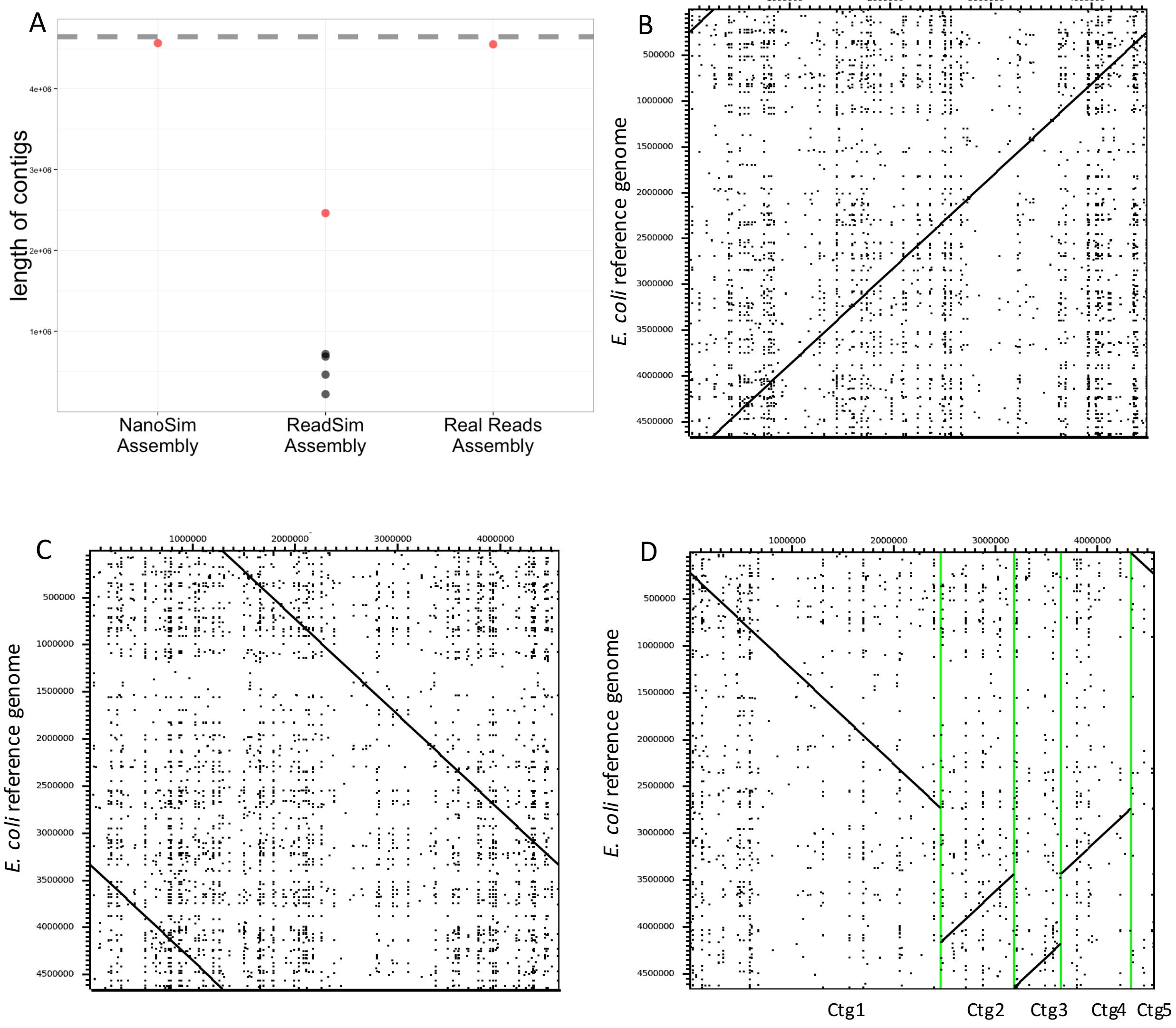

Click here to access/download
Supplementary Material
Supplementary method tables and figures_V2.pdf

Reference PDF marked changes

Click here to access/download
Supplementary Material
Reference PDF marked.pdf

Reference PDF

Click here to access/download
Supplementary Material
Reference PDF.pdf

Response to reviewers
Click here to download Personal Cover Response2Reviewers.pdf

Dear Dr. Edmunds,

Thank you for your consideration of our manuscript. We appreciate the thorough and constructive comments we received from you and our reviewers. In our revised submission, we have addressed the concerns raised, as outlined below, and edited the manuscript accordingly.

Sincerely,

Chen Yang / Inanc Birol
Genome Sciences Centre
British Columbia Cancer Agency

REVIEWER: 1

Major:

1. The authors use mixture models to model sequencing errors (page 2, lines 58-63). However, it is not clear in the manuscript how the models are learnt, ie, how the parameters are determined from real data. Furthermore, of description of the Markov chain and its associate properties (such as "transitional probability between two consecutive errors", "interarival time") is rather superficial. I believe these are the core of the tool and hence need to be discussed in more details.

Response: We appreciate the Reviewer's suggestion, and added detailed discussion about the model fitting and Markov chain building in the second paragraph on page 3 in the main text, as well as the supplementary method section page 2.

2. In the comparison section with ReadSim, I am not sure how the author ran ReadSim (I do not find what parameters were used). It appears that ReadSim simulated data closely similar to the E. coli R7.3 but not other datasets. Does it mean the parameters of ReadSim were tuned for R7.3 but not for other chemistry? Nanosim used the error profiles specific to each chemistry, and hence it is expected that its data were more similar to every dataset tested. I am curious to see how ReadSim performs on the error profiles learnt by Nanosim -- ReadSim may not accept the full profiles as Nanosim, but I noticed that it can take the error rates.

Response: We thank the Reviewer for this comment, and now state the parameters used in ReadSim clearly. We have also optimized the parameters for each dataset, including error rate, read length and sequencing technology. We now include a description of the parameters used in supplementary method page 3. Regarding the *E. coli* R7.3 dataset, only the full read length distribution is recovered well by ReadSim, while the lengths of unaligned regions on each read and the alignment ratio are not.

This is because ReadSim assumes uniform distribution of errors, and does not account for flanking head and tail regions of each read.

Minor point:

1. Now that there are several R9 datasets available, I am wondering if the authors can make available some training profile for R9 chemistry.

Response: Thank you for mentioning the R9 chemistry. We added the performance of NanoSim on R9 chemistry 2D dataset and R9 chemistry 1D rapid kit dataset to our supplementary material (supplementary figure S11, S12). We also uploaded the profiles to our ftp site for users to download.

REVIEWER: 2

1. The authors acknowledge that k-mer over and under representation is a known feature of nanopore data and attempt to resolve the most significant of these issues, that of homopolymer deficiency, by collapsing all homopolymers >n into a preset n-mer. This method seems somewhat simplistic, it may be better to include a stochastic element in the final n-mer length determination.

Response: We appreciate the Reviewer's valuable suggestion. To our knowledge, the HMM used in older Metrichor versions could not register self-transitions (such as AAAAA -> AAAAA) as the current signals are identical by definition. Homopolymer sequences are compressed into n-mer during basecalling, as their length are undetectable. NanoSim mimics this process in the last step of simulation, before which some homopolymers >n are already broken down by introduced errors. Aside from one special case mentioned below, we did not detect any homopolymers longer than 5 in all datasets (including the newly analyzed R9 1D reads), and thus this option is still a valuable option to more accurately simulate Oxford Nanopore reads. Admittedly, this method is over simplistic, because we noticed that sequencing or basecalling errors occur more often in homopolymer regions, including k-mers that contain a 4-mer and 3-mer homopolymer sequence. We tried to model this phenomenon, but did not observe a strong correlation between the lengths in the Reference compared to that of experimental reads. Therefore, adding a stochastic element becomes problematic. We added a few sentences in the manuscript on page 3 paragraph 6 to discuss this limitation.

The R9 2D dataset is an exception because the basecalling algorithm in Metrichor is changed to recurrent neural network. This dataset does not have a homopolymer under-representation problem, but instead, we noticed that it has the opposite problem; this dataset seems to have long homopolymers that don't exist in the reference genome. We

suspect this dataset may not be representative of the R9 chemistry and the underlying mechanism is not well-studied. We could not yet confidently produce a model for this phenomenon.

2. The authors also indicated that they do not have a 1D specific model but this tool should work the same for 1D reads. The authors have not shown any data to support this assertion an I am not confident that this simulation would work just as well for 1D reads. The statement that 2D reads are more accurate is fundamentally correct but neglects the fact that the error profile can be quite different between 1D and 2D reads. With the release of the 1D rapid kit and and the 1D ligation kit, 1D reads are becoming more and more important for ONT and neglecting them could be a serious failing. At the very minimum the authors should show how well their simulated reads perform in both a 1D and 2D context. Preferably, however, the authors should consider model parameters tuned to both 1D and 2D reads.

Response: We understand the Reviewer's concern and acknowledge the importance of 1D reads. In our revision, we benchmarked NanoSim on R9 chemistry 2D reads and 1D rapid kit and generated error profiles for users to download. Our results show that our model remains valid for these data types, as seen in supplementary figure S11, S12. Unfortunately, we did not find any publicly available dataset generated using the 1D ligation kit.
