# Supplementary material for: NanoSim: nanopore sequence read simulator based on statistical characterization
Source: Gigascience. 2017 Feb 24;6(4):1–6. doi: 10.1093/gigascience/gix010 (PMC5530317; doi:10.1093/gigascience/gix010)
Supplement: Supplemental material [file gix010_Supp.pdf]

Supplementary material for

NanoSim: nanopore sequence read simulator based on statistical characterization

Chen Yang[1,2], Justin Chu[1,2], René L Warren[2], and Inanç Birol[2,3,4, *]

[1]Faculty of Science, University of British Columbia, Vancouver, Canada,

[2]Genome Science Centre, British Columbia Cancer Agency, Vancouver, Canada,

[3]Department of Medical Genetics, University of British Columbia, Vancouver, Canada,

[4]School of Computing Science, Simon Fraser University, Burnaby, Canada.

Supplementary method section

Statistical test

In this paper, all statistical tests that decide the goodness of fit and the similarity between two samples are performed via Kolmogorov–Smirnov test (K-S test).

As a nonparametric statistical test, K-S test qualifies the distance between the empirical cumulative distribution function (ECDF) of the sample and the cumulative distribution function (CDF) of the reference distribution, or between the empirical distribution functions of two samples. The null hypothesis is that the sample is drawn from the reference distribution or samples are drawn from the same distribution, respectively. A large p-value (> 0.05) fails to reject the null hypothesis, and indicates the sample and the reference distribution or the two samples are statistically indistinguishable.

Modelling stage

The error models are determined by an R script. The parameters of mismatch model are estimated using maximum likelihood estimation (MLE) from the `stat4` package. The parameters of Indel models are estimated using grid search instead of MLE (for improved runtime). The goal is to minimize the objective function, namely the maximum difference between the ECDF and fitted CDF.

We used Markov chain to model (a) the error types and (b) the length of matched base calls. The transition matrices are learnt empirically. For the error type Markov chain, each state is one error type and there are three types: mismatch, insertion, and deletion. The match events are classified into equal-sized bins and each bin is a state in the match length Markov chain.

ReadSim parameters

We tuned all parameter combinations possible for `.readsim.py sim`, including:

`–rev_strd on`: This is turned on to create backward strands as well as forward strands

`–tech nanopore`: This parameter is used to tell ReadSim we are simulating ONT reads

`–read_mu`: The average read length is calculated for each dataset and provided to ReadSim

`–cov_mu`: The overall coverage for simulation is 35X, and 20,000 reads were randomly sampled from all simulated reads

`–err_sub_mu`: The average substitution rate is calculated for each dataset and provided to ReadSim

`–err_in_mu`: The average insertion rate is calculated for each dataset and provided to ReadSim

`–err_del_mu`: The average deletion rate is calculated for each dataset and provided to ReadSim

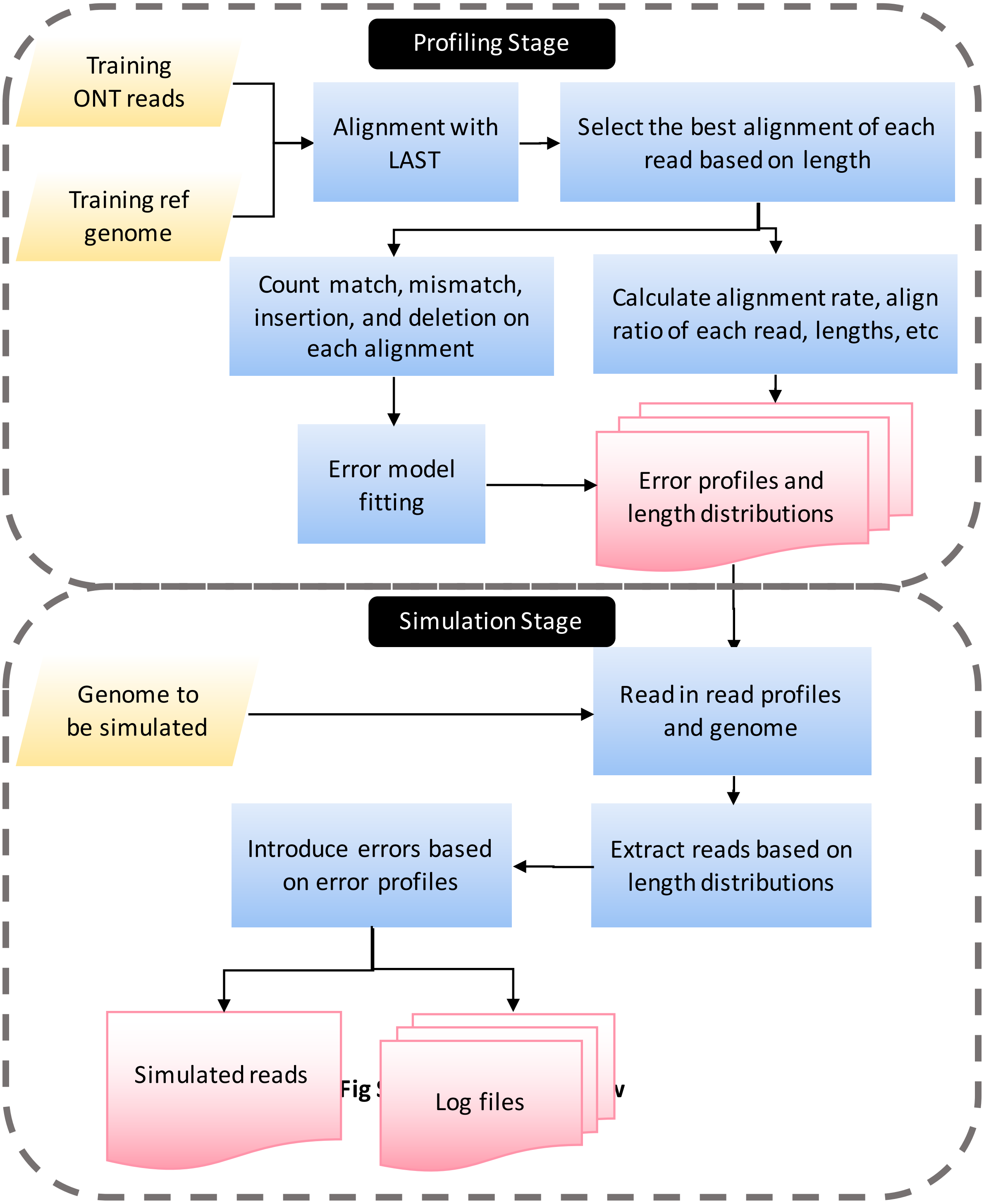

Fig S1. Flowchart of the Nanosim profiling and simulation stages.

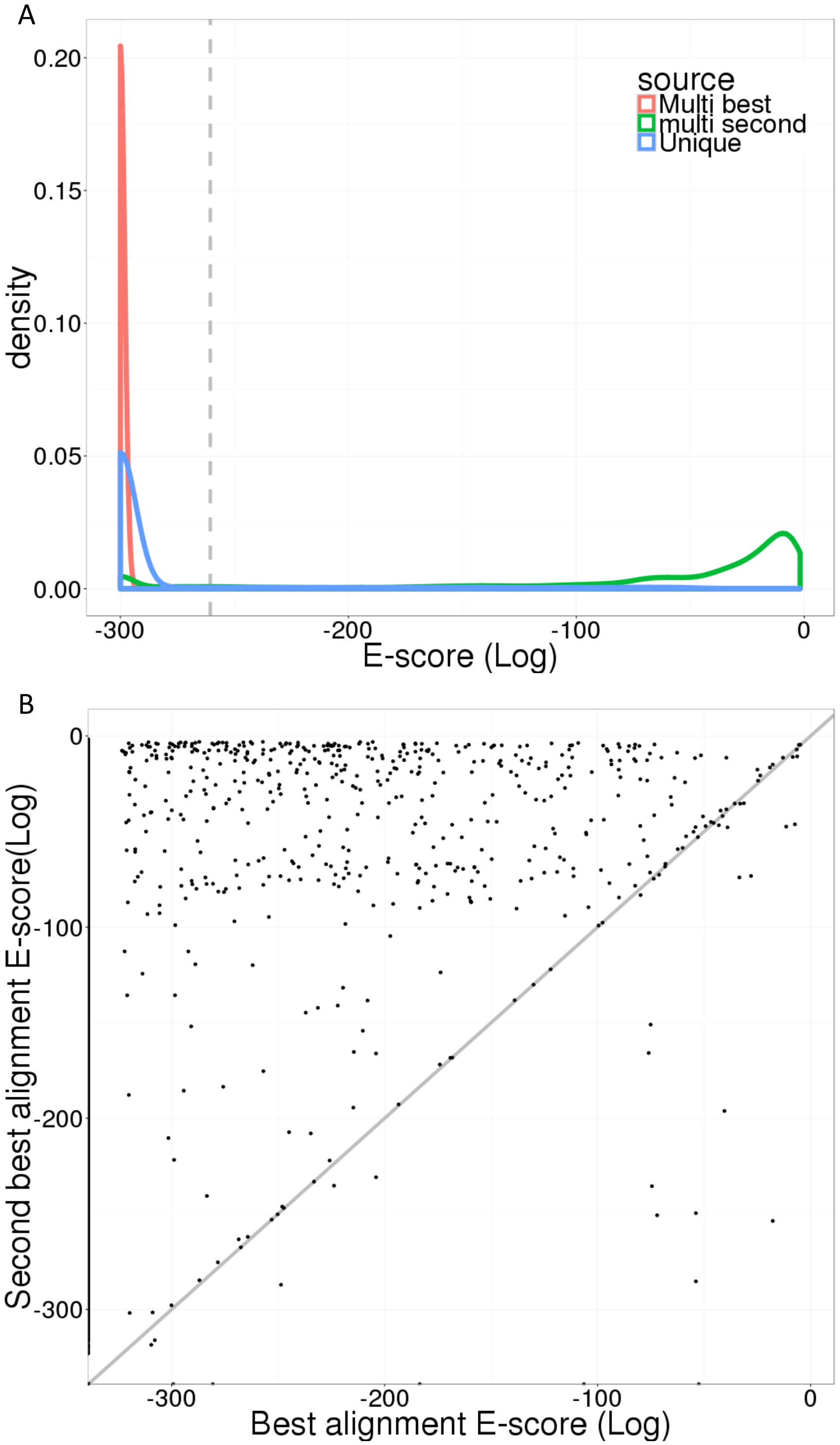

Fig S2. LAST alignment performance. (A) The best alignment of each read is chosen based on length and all best alignments have E-score lower than 3e-28. For all best and second best alignments that have E-score smaller than 2.65e-261, the second best alignment for all multi-aligned reads comprise 6.33%. (B) For multi-aligned reads, the E-score of the best alignment is generally lower than the second best, only a fraction of 0.1% second best alignments have a lower E-score than the best ones.

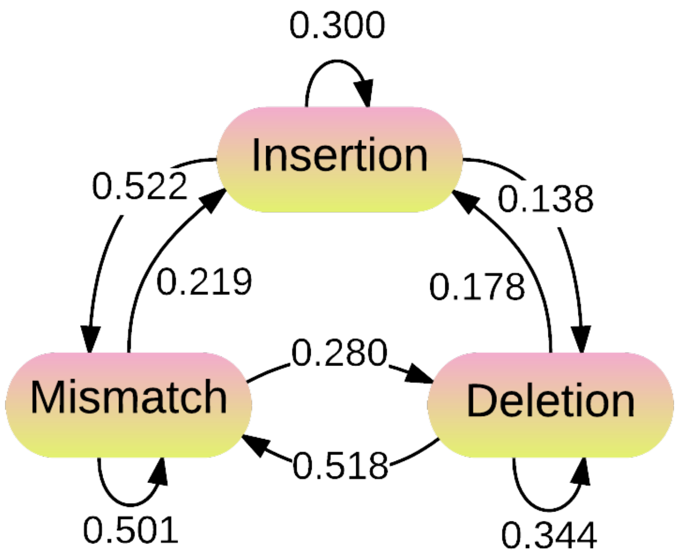

Fig S3. Transitional probabilities among different error type for *E. coli* R7 dataset. The probability of the first error type is not shown here.

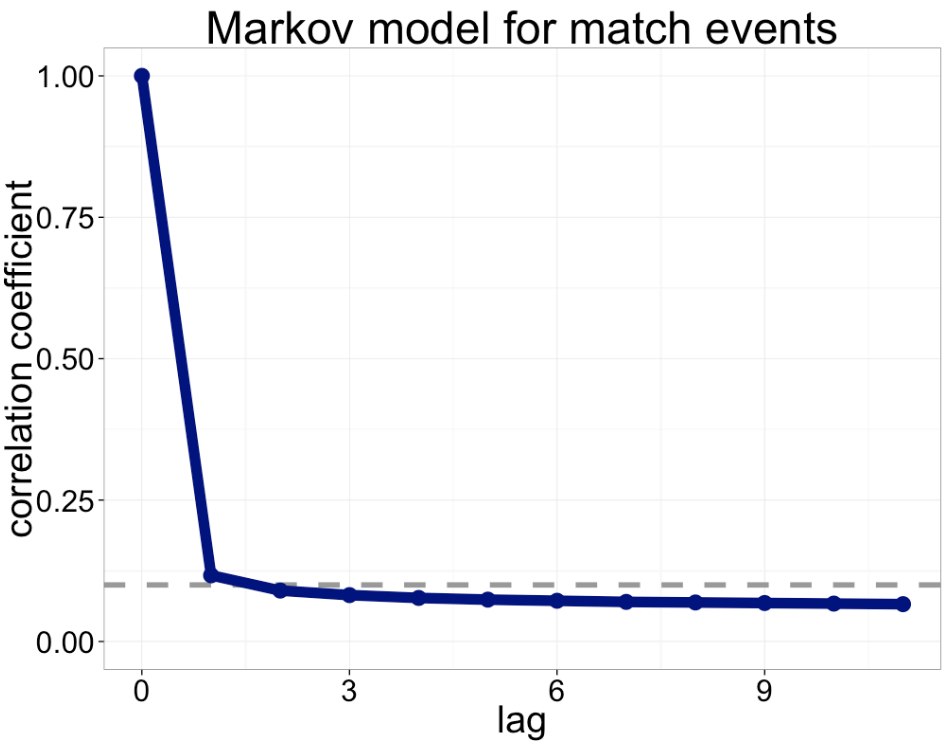

Fig S4. Auto-correlation of match events for *E. coli R7* dataset. The correlation coefficient of match events between time 0 and 1 is 0.117, suggesting the length of the previous correct base calls affects the length of the next. The coefficient drops below 0.1 at lag 2 and keeps decreasing.

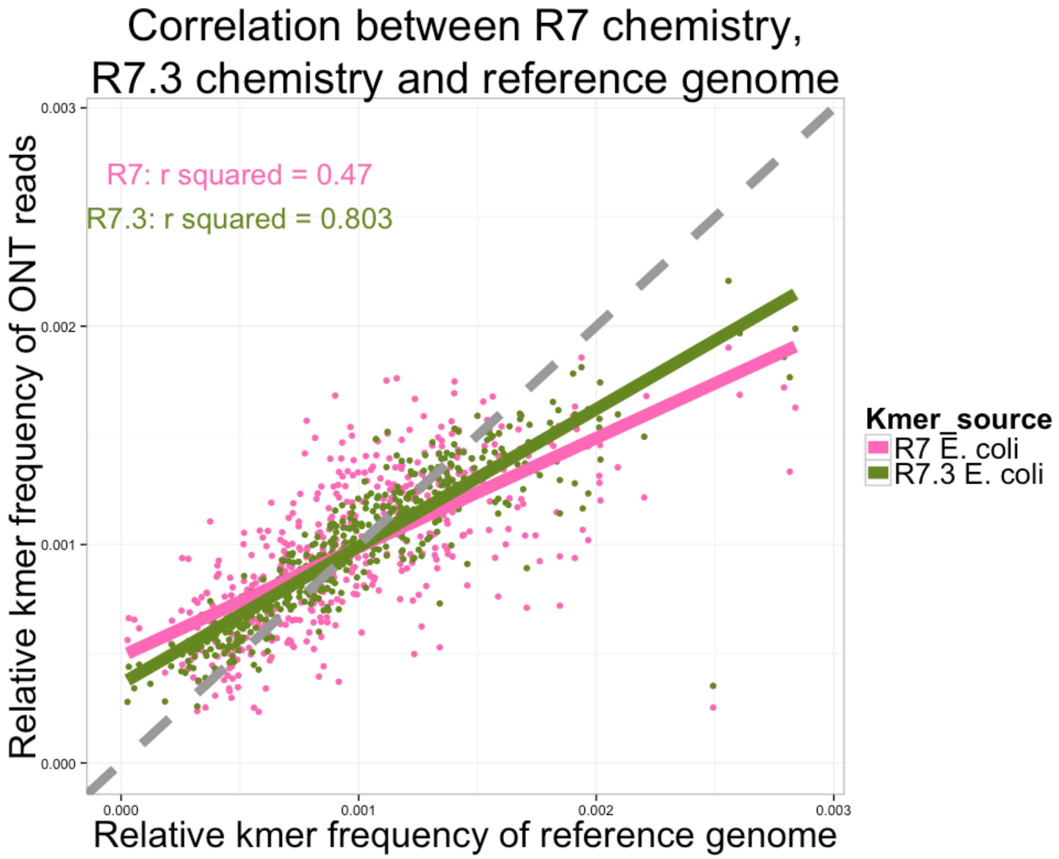

Fig S5. k-mer bias of *E. coli* R7 and R7.3 datasets. The relative 5-mer frequency of R7.3 chemistry has a stronger correlation with the reference genome than R7 chemistry.

Table S1. Mixture model parameters for mismatch. $P_m \sim \alpha$ Poisson (λ) + ($1 - \alpha$) Geometric (p)

Dataset	λ	p	α
E. coli R7	0.5339	0.7192	0.2325
E. coli R7.3	0.4673	0.7193	0.2930
E. coli UCSC	0.3971	0.7211	0.3705
E. coli R9 1D	0.1674	0.7060	0.1489
E. coli R9 2D	0.1761	0.6943	0.1239
yeast	0.4345	0.6973	0.2715

Table S2. Mixture model parameters for insertion. $P_i \sim \alpha$ Weibull (λ, κ) + ($1 - \alpha$) Geometric (p)

Dataset	λ	κ	p	α
E. coli R7	0.9571	0.9797	0.3955	0.9031
E. coli R7.3	1.1381	1.2183	0.4704	0.6023
E. coli UCSC	1.1810	1.3406	0.5267	0.5043
E. coli R9 1D	1.2790	1.5192	0.5292	0.5233
E. coli R9 2D	1.3019	1.1863	0.3263	0.6149
yeast	1.180	1.3021	0.4816	0.4880

Table S3. Mixture model parameters for deletion. $P_d \sim \alpha$ Weibull (λ, κ) + ($1 - \alpha$) Geometric (p)

Dataset	λ	κ	p	α
E. coli R7	1.0289	0.9923	0.4071	0.8548
E. coli R7.3	1.0972	1.2393	0.5523	0.5765
E. coli UCSC	1.2737	1.4084	0.5451	0.5006
E. coli R9 1D	1.2640	1.2805	0.4600	0.5640
E. coli R9 2D	1.0744	1.3226	0.4346	0.4478
yeast	0.9995	0.9899	0.2559	0.9571

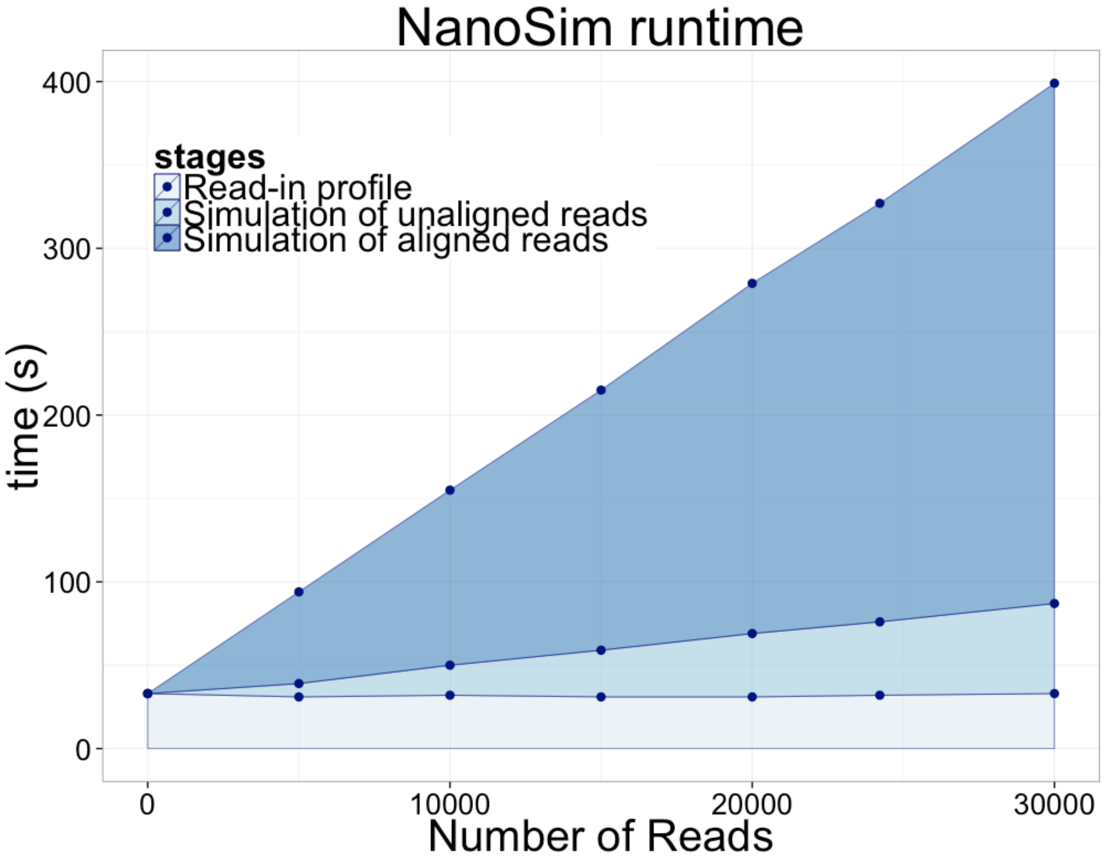

Fig S6. Runtime of NanoSim simulation stage on *E. coli* reference genome.

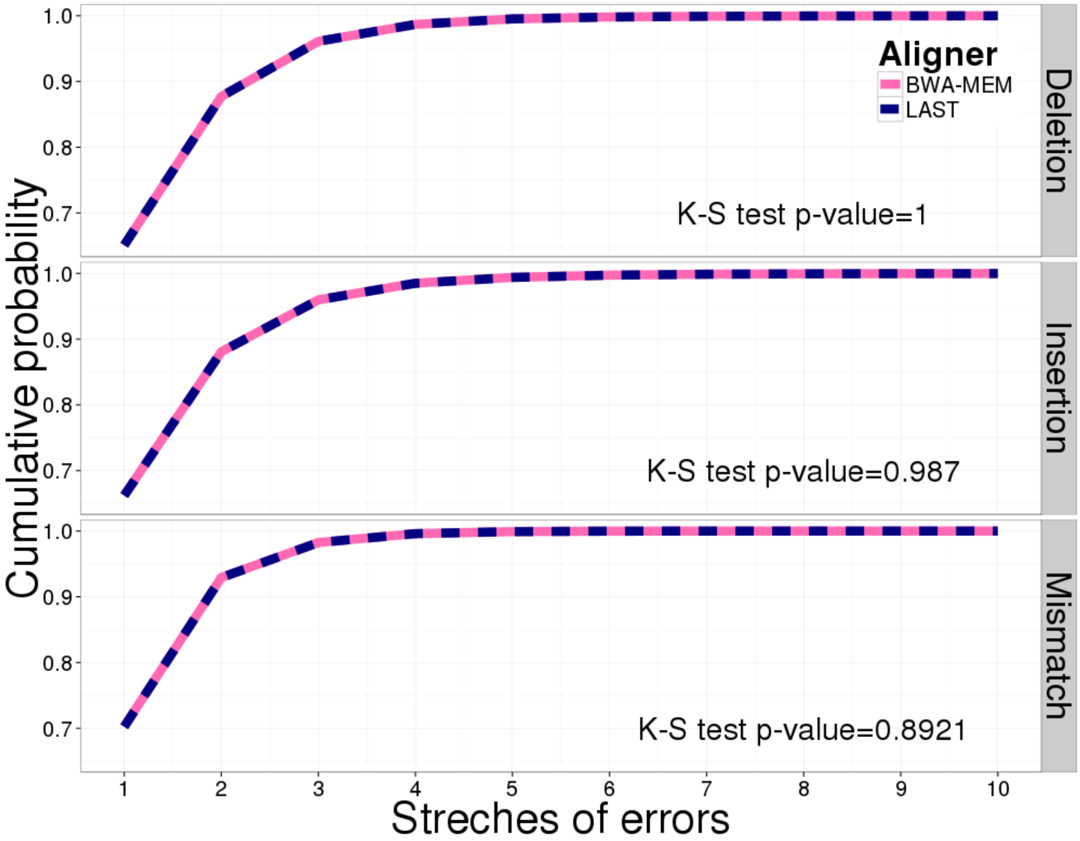

Fig S7. Error models derived from different aligners for *E. coli* UCSC dataset.

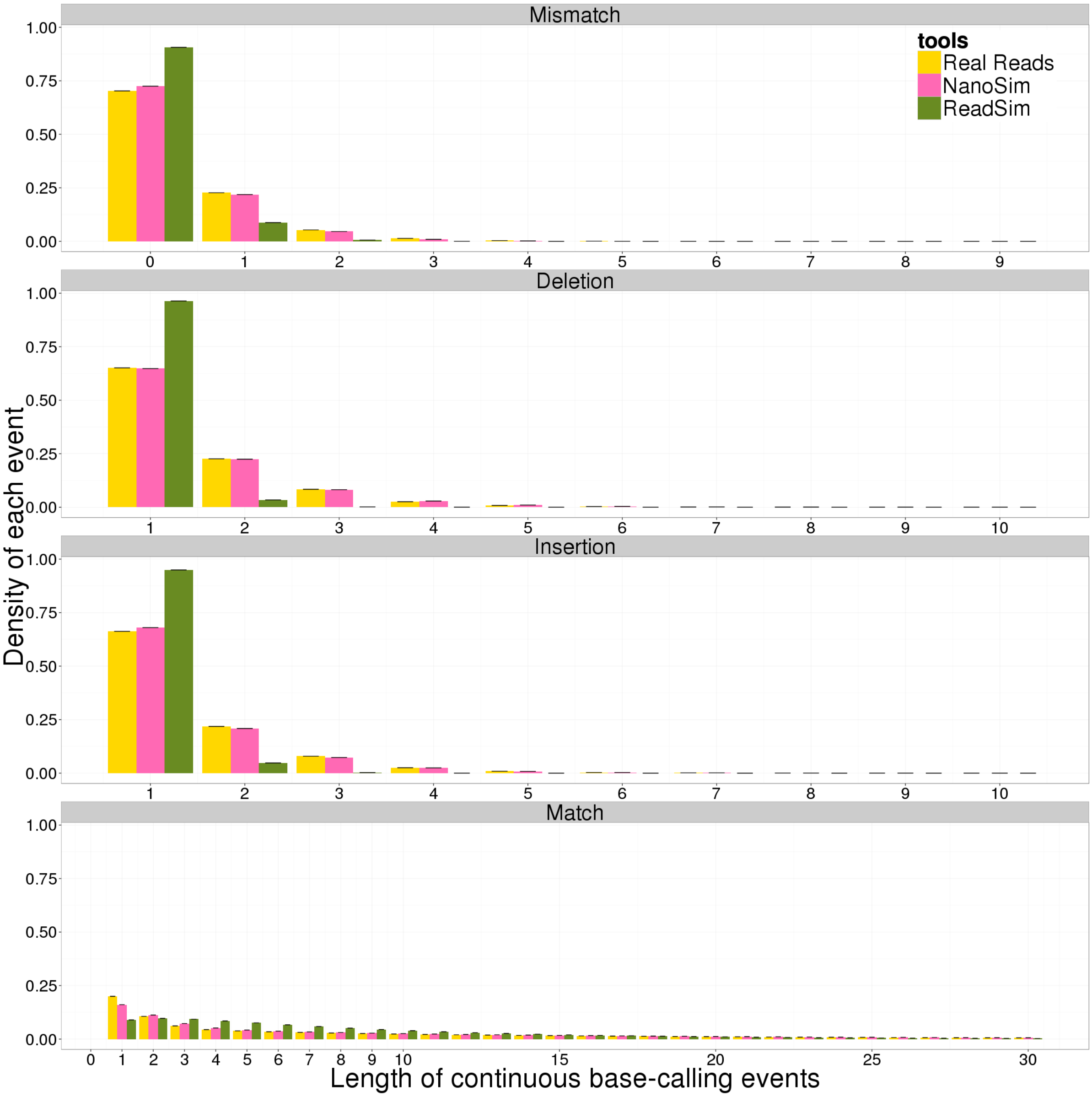

Fig S8. NanoSim simulation reads compared with *E. coli* UCSC experimental data and ReadSim simulated reads. Probability density bar plot plot of each error and matched base calls. The error bar is generated based on standard error.

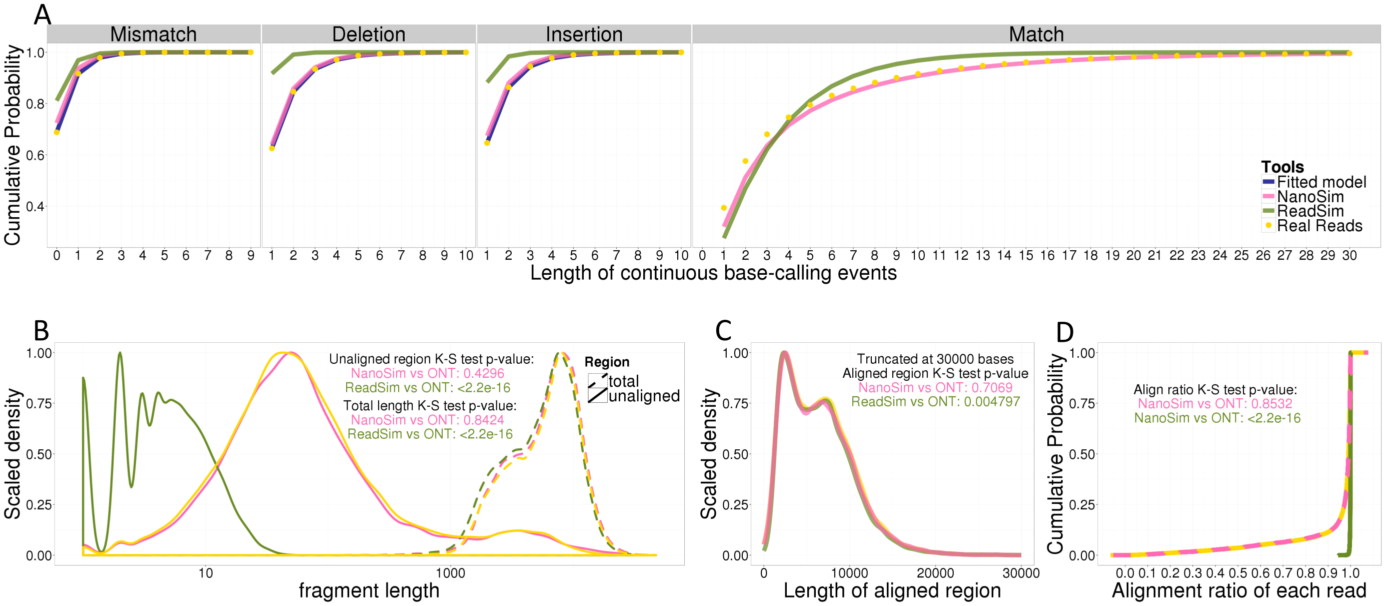

Fig S9. NanoSim simulation results compared with *E. coli* R7 experimental reads and ReadSim simulated reads. (A) The four plots on the upper panel are cumulative distribution plots of error match events and error events. (B) The length density plot of unaligned regions and total read lengths of aligned reads. (C) The length density plot of aligned regions on each read. (D) The cumulative density plot of alignment ratio of each read.

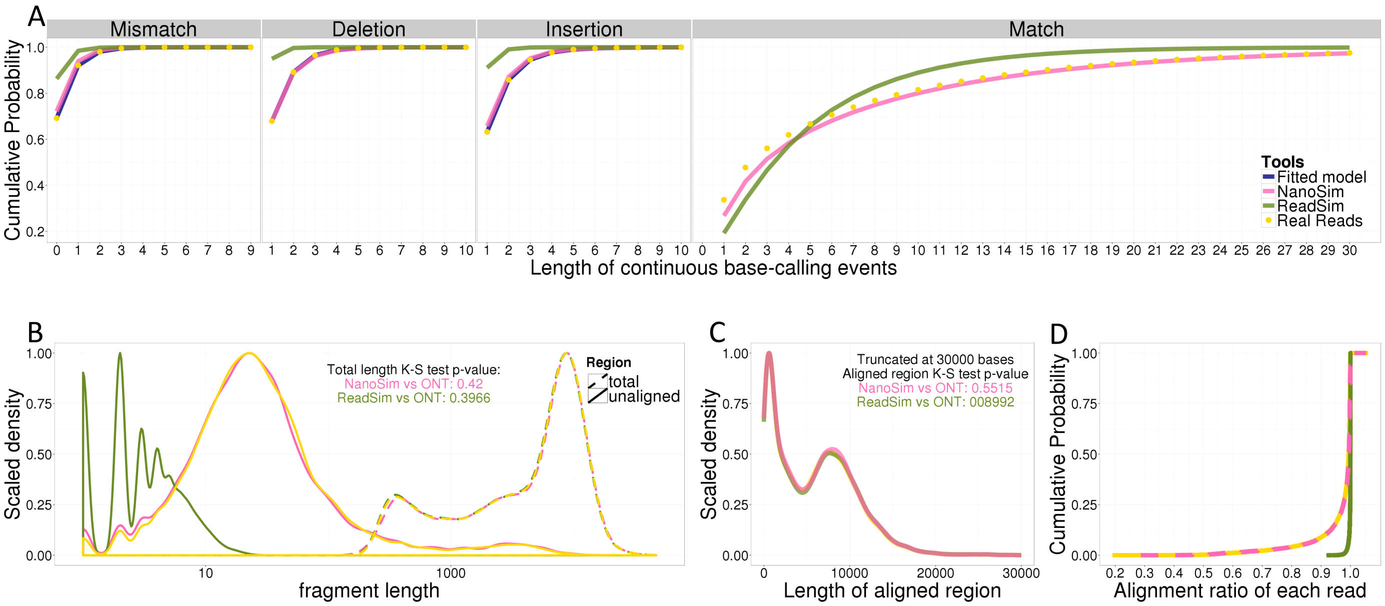

Fig S10. NanoSim simulation results compared with *E. coli* R7.3 experimental reads and ReadSim simulated reads. (A) The four plots on the upper panel are cumulative distribution plots of error match events and error events. (B) The length density plot of unaligned regions and total read length of aligned reads. (C) The length density plot of aligned regions on each read. (D) The cumulative density plot of alignment ratio of each read.

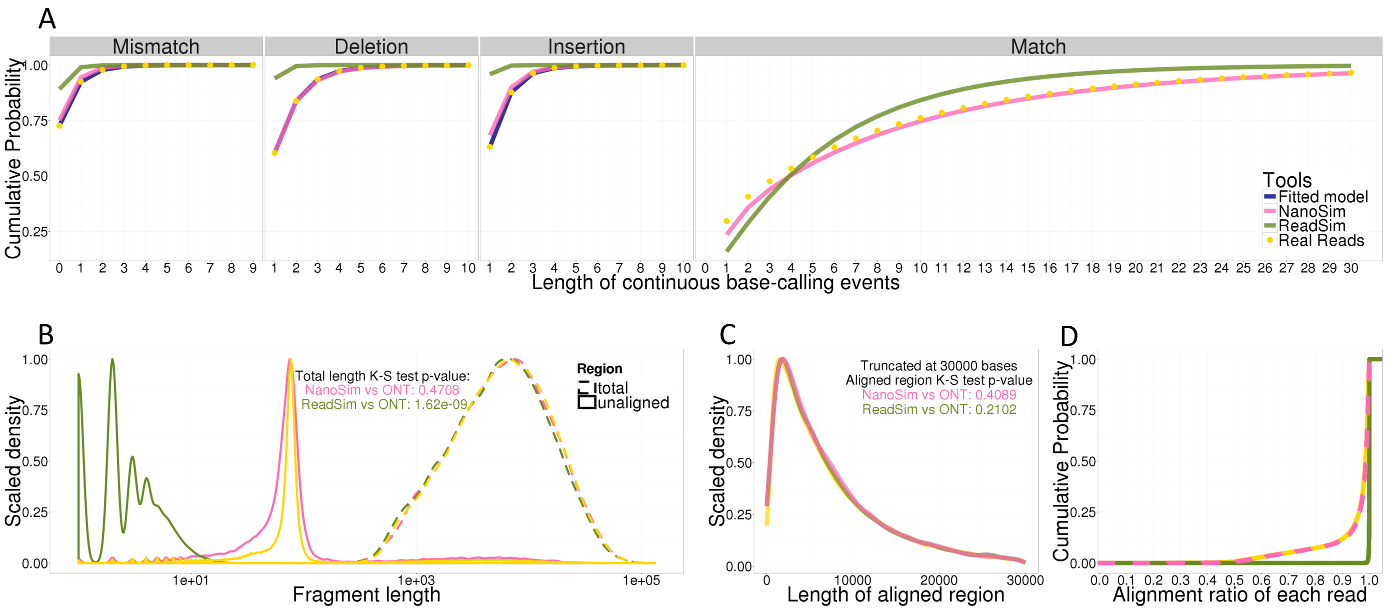

Fig S11. NanoSim simulation results compared with *E. coli* R9 1D experimental reads and ReadSim simulated reads. (A) The four plots on the upper panel are cumulative distribution plots of error match events and error events. (B) The length density plot of unaligned regions and total read lengths of aligned reads. (C) The length density plot of aligned regions on each read. (D) The cumulative density plot of alignment ratio of each read.

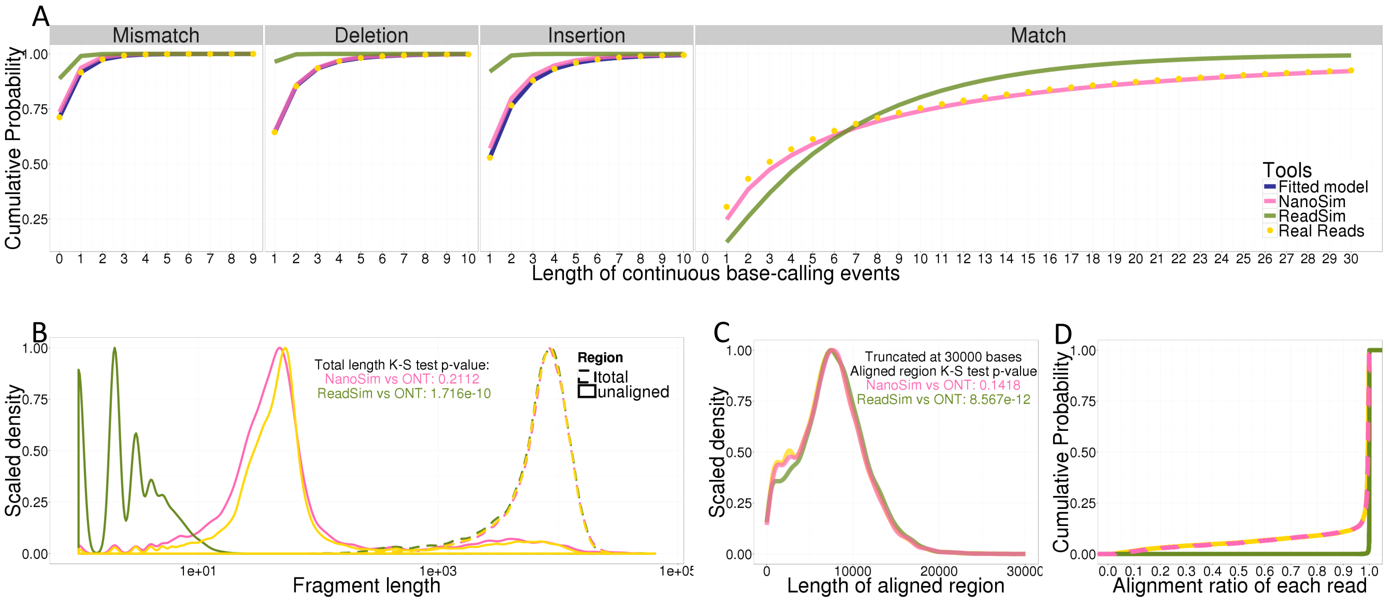

Fig S12. NanoSim simulation results compared with *E. coli* R9 2D experimental reads and ReadSim simulated reads. (A) The four plots on the upper panel are cumulative distribution plots of error match events and error events. (B) The length density plot of unaligned regions and total read length of aligned reads. (C) The length density plot of aligned regions on each read. (D) The cumulative density plot of alignment ratio of each read.

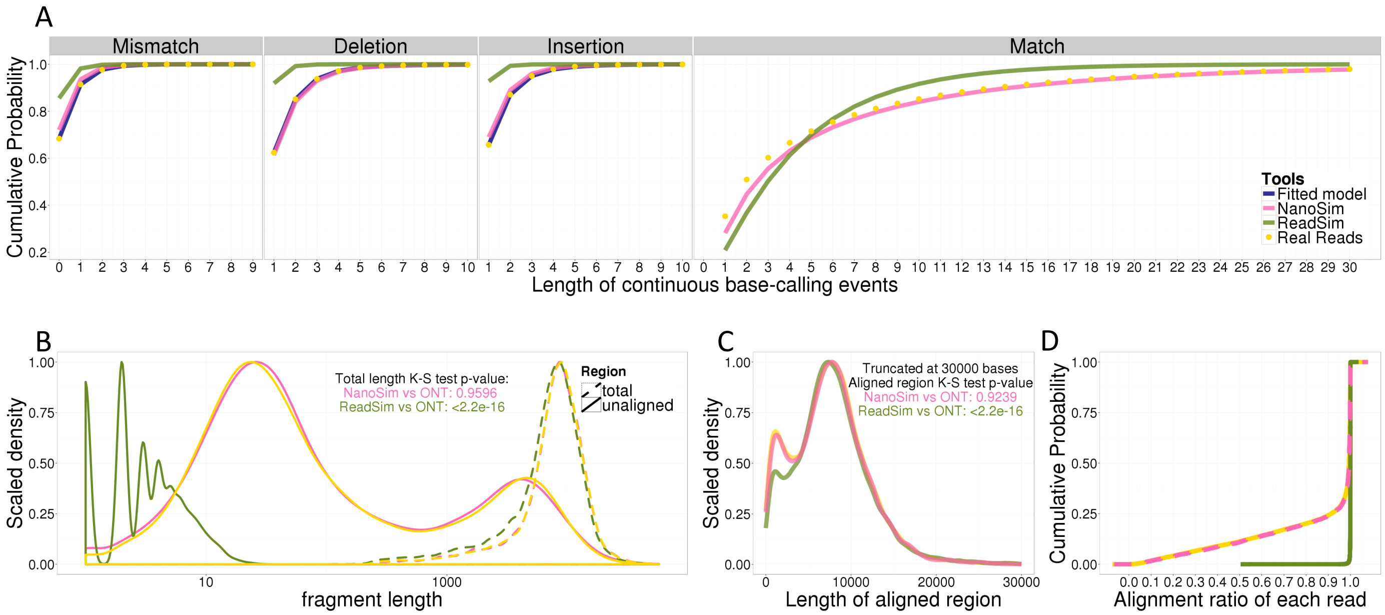

Fig S13. NanoSim simulation results compared with yeast experimental reads and ReadSim simulated reads. (A) The four plots on the upper panel are cumulative distribution plots of error match events and error events. (B) The length density plot of unaligned regions and total read lengths of aligned reads. (C) The length density plot of aligned regions on each read. (D) The cumulative density plot of alignment ratio of each read.
