# Supplementary material for: NanoSim: nanopore sequence read simulator based on statistical characterization
Source: Gigascience. 2017 Feb 24;6(4):1–6. doi: 10.1093/gigascience/gix010 (PMC5530317; doi:10.1093/gigascience/gix010)
Supplement: Response_to_Reviewers_Comments_Revision_1.pdf [file gix010_Response_to_Reviewers_Comments_Revision_1.pdf]

Dear Dr. Edmunds,

Thank you and our reviewers for your consideration of our manuscript. It is with excitement that we resubmit to you a revised version of manuscript “NanoSim: nanopore sequence read simulator based on statistical characterization”. In our revised submission, we have addressed the concerns raised by Reviewer 2 and edited the manuscript accordingly.

Sincerely,

Chen Yang / Inanc Birol
Genome Sciences Centre
British Columbia Cancer Agency

REVIEWER: 2
The authors have adequately and appropriately addressed my concerns; however I would like to see a citation for the statement on page 3 line 57-59. 2D reads from R9 chemistry does not have homopolymer underrepresentation problem, [sic] which is interpreted by a new basecalling algorithm using recurrent neural next work.

Response: Thanks for pointing this out. In the previous version, we stated that 2D reads from R9 chemistry does not have homopolymers underrepresentation problem, based on our analysis. We reworded paragraph 6 on page 3 in the revision to make it clear. We also added a citation to the statement about the recurrent neural network.
