# Supplementary material for: NanoSim: nanopore sequence read simulator based on statistical characterization
Source: Gigascience. 2017 Feb 24;6(4):1–6. doi: 10.1093/gigascience/gix010 (PMC5530317; doi:10.1093/gigascience/gix010)
Supplement: Response_to_reviewer_comments_Original_Submission.pdf [file gix010_Response_to_reviewer_comments_Original_Submission.pdf]

Dear Dr. Edmunds,

Thank you for your consideration of our manuscript. We appreciate the thorough and constructive comments we received from you and our reviewers. In our revised submission, we have addressed the concerns raised, as outlined below, and edited the manuscript accordingly.

Sincerely,

Chen Yang / Inanc Birol
Genome Sciences Centre
British Columbia Cancer Agency

REVIEWER: 1
Major:
1. The authors use mixture models to model sequencing errors (page 2, lines 58-63). However, it is not clear in the manuscript how the models are learnt, ie, how the parameters are determined from real data. Furthermore, of description of the Markov chain and its associate properties (such as "transitional probability between two consecutive errors", "interarival time") is rather superficial. I believe these are the core of the tool and hence need to be discussed in more details.
Response: We appreciate the Reviewer’s suggestion, and added detailed discussion about the model fitting and Markov chain building in the second paragraph on page 3 in the main text, as well as the supplementary method section page 2.
2. In the comparison section with ReadSim, I am not sure how the author ran ReadSim (I do not find what parameters were used). It appears that ReadSim simulated data closely similar to the E. coli R7.3 but not other datasets. Does it mean the parameters of ReadSim were tuned for R7.3 but not for other chemistry? Nanosim used the error profiles specific to each chemistry, and hence it is expected that its data were more similar to every dataset tested. I am curious to see how ReadSim performs on the error profiles learnt by Nanosim -- ReadSim may not accept the full profiles as Nanosim, but I noticed that it can take the error rates.
Response: We thank the Reviewer for this comment, and now state the parameters used in ReadSim clearly. We have also optimized the parameters for each dataset, including error rate, read length and sequencing technology. We now include a description of the parameters used in supplementary method page 3. Regarding the E. coli R7.3 dataset, only the full read length distribution is recovered well by ReadSim, while the lengths of unaligned regions on each read and the alignment ratio are not. This is because ReadSim assumes uniform distribution of errors, and does not account for flanking head and tail regions of each read.
Minor point:
1. Now that there are several R9 datasets available, I am wondering if the authors can make available some training profile for R9 chemistry.
Response: Thank you for mentioning the R9 chemistry. We added the performance of NanoSim on R9 chemistry 2D dataset and R9 chemistry 1D rapid kit dataset to our supplementary material (supplementary figure S11, S12). We also uploaded the profiles to our ftp site for users to download.

REVIEWER: 2
1. The authors acknowledge that k-mer over and under representation is a known feature of nanopore data and attempt to resolve the most significant of these issues, that of homopolymer deficiency, by collapsing all homopolymers >n into a preset n-mer. This method seems somewhat simplistic, it may be better to include a stochastic element in the final n-mer length determination.
Response: We appreciate the Reviewer’s valuable suggestion. To our knowledge, the HMM used in older Metrichor versions could not register self-transitions (such as AAAAA -> AAAAA) as the current signals are identical by definition. Homopolymer sequences are compressed into n-mer during basecalling, as their length are undetectable. NanoSim mimics this process in the last step of simulation, before which some

homopolymers >n are already broken down by introduced errors. Aside from one special case mentioned below, we did not detect any homopolymers longer than 5 in all datasets (including the newly analyzed R9 1D reads), and thus this option is still a valuable option to more accurately simulate Oxford Nanopore reads. Admittedly, this method is over simplistic, because we noticed that sequencing or basecalling errors occur more often in homopolymer regions, including k-mers that contain a 4-mer and 3-mer homopolymer sequence. We tried to model this phenomenon, but did not observe a strong correlation between the lengths in the Reference compared to that of experimental reads. Therefore, adding a stochastic element becomes problematic. We added a few sentences in the manuscript on page 3 paragraph 6 to discuss this limitation. The R9 2D dataset is an exception because the basecalling algorithm in Metrichor is changed to recurrent neural network. This dataset does not have a homopolymer under-representation problem, but instead, we noticed that it has the opposite problem; this dataset seems to have long homopolymers that don't exist in the reference genome. We suspect this dataset may not be representative of the R9 chemistry and the underlying mechanism is not well-studied. We could not yet confidently produce a model for this phenomenon.

2. The authors also indicated that they do not have a 1D specific model but this tool should work the same for 1D reads. The authors have not shown any data to support this assertion an I am not confident that this simulation would work just as well for 1D reads. The statement that 2D reads are more accurate is fundamentally correct but neglects the fact that the error profile can be quite different between 1D and 2D reads. With the release of the 1D rapid kit and and the 1D ligation kit, 1D reads are becoming more and more important for ONT and neglecting them could be a serious failing. At the very minimum the authors should show how well their simulated reads perform in both a 1D and 2D context. Preferably, however, the authors should consider model parameters tuned to both 1D and 2D reads.

Response: We understand the Reviewer's concern and acknowledge the importance of 1D reads. In our revision, we benchmarked NanoSim on R9 chemistry 2D reads and 1D rapid kit and generated error profiles for users to download. Our results show that our model remains valid for these data types, as seen in supplementary figure S11, S12. Unfortunately, we did not find any publicly available dataset generated using the 1D ligation kit.
