# Supplementary material for: NanoSim: nanopore sequence read simulator based on statistical characterization
Source: Gigascience. 2017 Feb 24;6(4):1–6. doi: 10.1093/gigascience/gix010 (PMC5530317; doi:10.1093/gigascience/gix010)
Supplement: Reviewer_1_Report_(Original_Submission).pdf [file gix010_Reviewer_1_Report_(Original_Submission).pdf]

Reviewer Report

Title: "NanoSim: nanopore sequence read simulator based on statistical characterization"

Version: Original Submission **Date:** 11/13/2016

Reviewer name: Minh Duc Cao

Reviewer Comments to Author:

Yang et al present a method for simulation of nanopore sequencing data. The tool consists of two components: a profiler to learn error probabilities from a training data set, and a simulator to generate simulated data using the error profiles. In general, the method would be useful for practitioners working with nanopore sequencing data. I have the following concerns:

Major:

1. The authors use mixture models to model sequencing errors (page 2, lines 58-63). However, it is not clear in the manuscript how the models are learnt, ie, how the parameters are determined from real data. Furthermore, of description of the Markov chain and its associate properties (such as "transitional probability between two consecutive errors", "interarival time") is rather superficial. I believe these are the core of the tool and hence need to be discussed in more details.

2. In the comparison section with ReadSim, I am not sure how the author ran ReadSim (I do not find what parameters were used). It appears that ReadSim simulated data closely similar to the E. coli R7.3 but not other datasets. Does it mean the parameters of ReadSim were tuned for R7.3 but not for other chemistry? Nanosim used the error profiles specific to each chemistry, and hence it is expected that its data were more similar to every dataset tested. I am curious to see how ReadSim performs on the error profiles learnt by Nanosim -- ReadSim may not accept the full profiles as Nanosim, but I noticed that it can take the error rates

Minor point:

1. Now that there are several R9 datasets available, I am wondering if the authors can make available some training profile for R9 chemistry.

Level of Interest

Please indicate how interesting you found the manuscript: An article whose findings are important to those with closely related research interests

Quality of Written English

Please indicate the quality of language in the manuscript: Needs some language corrections before being published

Declaration of Competing Interests

Please complete a declaration of competing interests, considering the following questions:

- Have you in the past five years received reimbursements, fees, funding, or salary from an organisation that may in any way gain or lose financially from the publication of this manuscript, either now or in the future?
- Do you hold any stocks or shares in an organisation that may in any way gain or lose financially from the publication of this manuscript, either now or in the future?
- Do you hold or are you currently applying for any patents relating to the content of the manuscript?
- Have you received reimbursements, fees, funding, or salary from an organization that holds or has applied for patents relating to the content of the manuscript?
- Do you have any other financial competing interests?
- Do you have any non-financial competing interests in relation to this paper?

If you can answer no to all of the above, write 'I declare that I have no competing interests' below. If your reply is yes to any, please give details below.

I declare that I have no competing interests

I agree to the open peer review policy of the journal. I understand that my name will be included on my report to the authors and, if the manuscript is accepted for publication, my named report including any attachments I upload will be posted on the website along with the authors' responses. I agree for my report to be made available under an Open Access Creative Commons CC-BY license (http://creativecommons.org/licenses/by/4.0/). I understand that any comments which I do not wish to be included in my named report can be included as confidential comments to the editors, which will not be published.

I agree to the open peer review policy of the journal
