# Supplementary material for: NanoSim: nanopore sequence read simulator based on statistical characterization
Source: Gigascience. 2017 Feb 24;6(4):1–6. doi: 10.1093/gigascience/gix010 (PMC5530317; doi:10.1093/gigascience/gix010)
Supplement: Reviewer_2_Report_(Original_Submission).pdf [file gix010_Reviewer_2_Report_(Original_Submission).pdf]

Reviewer Report

Title: "NanoSim: nanopore sequence read simulator based on statistical characterization"Original Submission

Version: Original Submission **Date:** 11/29/2016

Reviewer name: Sara Goodwin

Reviewer Comments to Author:

This article outlines and method for generating simulated Oxford Nanopore (ONT) reads. The authors discuss the rational for why an ONT dedicated simulator is needed and what methods were employed to simulate the unique error profile of ONT data. Unlike other methods for simulating ONT reads which assume uniformly distributed errors, the authors attempt to model the non-uniform nature of ONT data.

The authors have shown improved performance of reads simulated in this manner compared to the ReadSim simulator. Despite the improvements, some potential deficiencies exist in the modeling approached used.

The authors acknowledge that k-mer over and under representation is a known feature of nanopore data and attempt to resolve the most significant of these issues, that of homopolymer deficiency, by collapsing all homopolymers >n into a preset n-mer. This method seems somewhat simplistic, it may be better to include a stochastic element in the final n-mer length determination.

The authors also indicated that they do not have a 1D specific model but this tool should work the same for 1D reads. The authors have not shown any data to support this assertion an I am not confident that this simulation would work just as well for 1D reads. The statement that 2D reads are more accurate is fundamentally correct but neglects the fact that the error profile can be quite different between 1D and 2D reads. With the release of the 1D rapid kit and and the 1D ligation kit, 1D reads are becoming more and more important for ONT and neglecting them could be a serious failing. At the very minimum the authors should show how well their simulated reads perform in both a 1D and 2D context. Preferably, however, the authors should consider model parameters tuned to both 1D and 2D reads.

On the whole this manuscript provides a tool that will be useful researchers working with nanopore data. In it's current form this tool will be useful for preliminary bench-marking studies. However substantial effort will need to be dedicated to both keeping up with the ever changing ONT chemistries and to more accurately represent the full repertoire of ONT reads.

Level of Interest

Please indicate how interesting you found the manuscript: An article whose findings are important to those with closely related research interests

Quality of Written English

Please indicate the quality of language in the manuscript: Acceptable

Declaration of Competing Interests

Please complete a declaration of competing interests, considering the following questions:

- Have you in the past five years received reimbursements, fees, funding, or salary from an organisation that may in any way gain or lose financially from the publication of this manuscript, either now or in the future?
- Do you hold any stocks or shares in an organisation that may in any way gain or lose financially from the publication of this manuscript, either now or in the future?
- Do you hold or are you currently applying for any patents relating to the content of the manuscript?
- Have you received reimbursements, fees, funding, or salary from an organization that holds or has applied for patents relating to the content of the manuscript?
- Do you have any other financial competing interests?
- Do you have any non-financial competing interests in relation to this paper?

If you can answer no to all of the above, write 'I declare that I have no competing interests' below. If your reply is yes to any, please give details below.

I declare that I have no competing interests

I agree to the open peer review policy of the journal. I understand that my name will be included on my report to the authors and, if the manuscript is accepted for publication, my named report including any attachments I upload will be posted on the website along with the authors' responses. I agree for my report to be made available under an Open Access Creative Commons CC-BY license (http://creativecommons.org/licenses/by/4.0/). I understand that any comments which I do not wish to be included in my named report can be included as confidential comments to the editors, which will not be published.

I agree to the open peer review policy of the journal
